# Performance Evaluation and Online Realization of Data-driven Normalization Methods Used in LC/MS based Untargeted Metabolomics Analysis

**DOI:** 10.1038/srep38881

**Published:** 2016-12-13

**Authors:** Bo Li, Jing Tang, Qingxia Yang, Xuejiao Cui, Shuang Li, Sijie Chen, Quanxing Cao, Weiwei Xue, Na Chen, Feng Zhu

**Affiliations:** 1 Innovative Drug Research and Bioinformatics Group, Innovative Drug Research Centre and School of Pharmaceutical Sciences, Chongqing University, Chongqing 401331, China; 2 College of Mathematics and Statistics, Chongqing University, Chongqing 401331, China

## Abstract

In untargeted metabolomics analysis, several factors (e.g., unwanted experimental & biological variations and technical errors) may hamper the identification of differential metabolic features, which requires the data-driven normalization approaches before feature selection. So far, ≥16 normalization methods have been widely applied for processing the LC/MS based metabolomics data. However, the performance and the sample size dependence of those methods have not yet been exhaustively compared and no online tool for comparatively and comprehensively evaluating the performance of all 16 normalization methods has been provided. In this study, a comprehensive comparison on these methods was conducted. As a result, 16 methods were categorized into three groups based on their normalization performances across various sample sizes. The *VSN*, the *Log Transformation* and the *PQN* were identified as methods of the best normalization performance, while the *Contrast* consistently underperformed across all sub-datasets of different benchmark data. Moreover, an interactive web tool comprehensively evaluating the performance of 16 methods specifically for normalizing LC/MS based metabolomics data was constructed and hosted at http://server.idrb.cqu.edu.cn/MetaPre/. In summary, this study could serve as a useful guidance to the selection of suitable normalization methods in analyzing the LC/MS based metabolomics data.

Metabolomics aims at characterizing metabolic biomarkers by analytically describing complex biological samples^1^. At present, the metabolomics based on liquid chromatography mass spectrometry (LC/MS) is capable of simultaneously monitoring thousands of metabolites in bio-fluid, cell and tissue, and is widely applied to various aspects of biomedical research. In particular, metabolomics analysis on LC/MS data can aid the choice of therapy^2^, provide powerful tools for drug discovery by revealing drug mechanism of actions and potential side effects^3^, and help to identify biomarkers^4^,^5^,^6^ of various diseases such as hepatocellular carcinoma (HCC)^7^, colorectal cancer^8^, insulin resistance^9^, and so on.

Several factors (e.g., unwanted experimental & biological variations and technical errors) may hamper the identification of differential metabolic profiles and effectiveness of metabolomics analysis (e.g., paired or nested studies)^10^,^11^,^12^,^13^,^14^. To remove specific types of unwanted variations, the signal drift correction (when quality control samples are available), the batch effect removal (when internal standards or quality control samples are available), and the scaling (not suitable when the self-averaging property does not hold) are adopted^13^. These commonly used strategies are generally grouped into two categories: (1) method-driven normalization approaches extrapolating external model that is based upon internal standards or quality control samples and (2) data-driven normalization approaches scaling or transforming metabolomics data^15^,^16^,^17^,^18^,^19^,^20^. As reported in Ejigu’s work, the method-driven strategies may not be practical due to several reasons, especially their unsuitability for treating untargeted metabolomics data, while data-driven ones are better choices for untargeted LC/MS based metabolomics data^15^. The capacities of 11 data-driven normalization methods (“normalization method” in short for the rest of this paper) for processing nuclear magnetic resonance (NMR) based metabolomics data were systematically compared^21^. Two methods (the *Quantile* and the *Cubic Splines*) were identified as the “best” performed normalization methods, while other two methods (the *Contrast* and the *Li-Wong*) could “hardly” reduce bias at all and could not improve the comparability between samples^21^. For gas chromatography mass spectrometry (GC/MS) based metabolomics, a comparative research on the performances of 8 normalization methods discovered two (the *Auto Scaling* and the *Range Scaling*) of “overall best performance”^12^. Similar to NMR and GC/MS, the LC/MS is one of the most popular sources of current metabolomics data, and it is of great importance to analyze the differential influence of those methods on LC/MS based data. Ejigu *et al*. measured the performance of 6 methods according to their “average metabolite specific coefficient of variation (CV)”^15^. The CV showed that the *Cyclic Loess* and the *Cubic Splines* performed “slightly better” than other methods, but no statistical difference among CVs of those methods was observed^15^.

For the past decade, no less than 16 methods have been developed for normalizing the LC/MS based metabolomics data^13^,^22^,^23^, some of which (e.g., the *VSN*^24^, the *Quantile*^25^, the *Cyclic Loess*^26^) are directly adopted from those previously used for processing transcriptomics data. Both metabolomics data and transcriptomics data are high-dimensional. However, the dimension of transcriptomics data can reach 10 thousands, while that of metabolomics data is about a few thousands. Moreover, unlike transcriptomics, correlation among metabolites identified from metabolomics data may not indicate a common biological function^27^. Apart from the above differences, there are significant similarities between two OMICs data: (1) right-skewed distribution^23^, (2) great data sparsity^28^, (3) substantial amount of noise^29^,^30^ and (4) significantly varied sample sizes^31^,^32^. Due to these similarities, it is feasible to apply some of the normalization methods used in transcriptomics data analysis to the metabolomics one.

Those 16 methods specifically normalizing LC/MS based metabolomics data can be classified into two groups^21^. Methods in group one (including the *Contrast Normalization*^33^, the *Cubic Splines*^34^, the *Cyclic Loess*^35^, the *Linear Baseline Scaling*^25^, the *MSTUS*^22^, the *Non-Linear Baseline Normalization*^36^, the *Probabilistic Quotient Normalization*^37^ and the *Quantile Normalization*^25^) aim at removing the unwanted sample-to-sample variations, while methods of the second group (including the *Auto Scaling*^38^, the *Level Scaling*^12^, the *Log Transformation*^39^, the *Pareto Scaling*^40^, the *Power Scaling*^41^, the *Range Scaling*^42^, the *VSN*^43^,^44^ and the *Vast Scaling*^45^) adjust biases among various metabolites to reduce heteroscedasticity. However, the performance and the sample size dependence of those methods widely adopted in current metabolomics studies (e.g., the *Pareto Scaling* and the *VSN*)^28^,^46^ have not yet been exhaustively compared in the context of LC/MS metabolomics data analysis.

Moreover, several comprehensive metabolomics pipelines are currently available online, where various normalization algorithms are integrated in as one step in their corresponding analysis chain. These online pipelines include the *MetaboAnalyst*^28^, the *Metabolomics Workbench*^47^, the *MetaDB*^48^, the *MetDAT*^49^, the *MSPrep*^50^, the *Workflow4Metabolomics*^51^ and the *XCMS online*^52^. Based on a comprehensive review, the number of normalization algorithms provided by the above pipelines varies significantly from 2 (the *Workflow4Metabolomics*) to 13 (the *MetaboAnalyst*). 6 out of those 7 pipelines only provide <50% of those 16 methods analyzed in this study. The *MetaboAnalyst* is the only pipeline offering 13 methods, but some methods reported as “well-performed” in LC/MS based metabolomics analysis (e.g., the *VSN* and the *PQN*)^28^,^37^,^46^ are not provided. The inadequate coverage of these methods may weaken the applicability range of those pipelines. Moreover, the suitability of a normalization method was reported to be greatly dependent on the nature of the analyzed data^53^, a comparative performance evaluation among methods is therefore essential to the determination of the most appropriate method for professional/inexperienced researchers. However, no comparative evaluation among those normalization methods was conducted in the above pipelines. So far, the *Normalyzer*^53^ is the only online tool offering comparative evaluation of 12 different normalization methods treating high-throughput OMICs data^53^. In particular, this tool accepted a variety of data types including metabolomics, proteomics, DNA microarray and the real-time polymerase chain reaction data^53^. However, since the *Normalyzer* was designed to process a wide range of OMICs, it did not cover 8 of those 16 methods specifically for LC/MS based metabolomics studies. Thus, it is in urgent need to construct a publicly available tool for comparatively and comprehensively evaluating the performances of methods used specifically for normalizing LC/MS based metabolomics data.

In this study, a comprehensive comparison on the normalization capacities of 16 methods was conducted. Firstly, the differential metabolic features selected based on each method were validated by a benchmark spike-in dataset and by experimentally validated markers. To further understand the influence of sample size on the method performance, 10 sub-datasets of various sample size were generated to evaluate the variation of normalization performance among 16 methods, and to categorize these methods into 3 groups (superior, good and poor performance group). Finally, a web-based tool used to comprehensively evaluate the performance of all 16 methods was constructed. In sum, this study could serve as valuable guidance to the selection of suitable normalization methods in analyzing the LC/MS based metabolomics data.

## Materials and Methods

### Benchmark datasets collection and sub-datasets generation

Five criteria were used to select datasets from the *MetaboLights* (http://www.ebi.ac.uk/metabolights/)^32^ in this study, which include: (1) data type set as “study”; (2) technology set as “mass spectrometry”; (3) organism set as “*homo sapiens*”; (4) study validation set as “fully validated”; (5) untargeted LC/MS based metabolomics data with >100 samples selected by manual literature and dataset reviews. Based on the above criteria, 4 benchmark datasets were collected for analysis, which include the positive (ESI+) and negative (ESI−) ionization modes of both MTBLS28^54^ and MTBLS17^55^. For MTBLS17, only the dataset of experiment 1 with >100 studied samples was included. For the remaining text of this paper, MTBLS17 was used to stand for the dataset of experiment 1 in Ressom’s work^55^. Both ESI+ and ESI− of MTBLS28 provided LC/MS based metabolomics profiles of 1,005 samples (469 lung cancer patients and 536 healthy individuals)^54^, and MTBLS17 ESI+ and ESI− gave profiles of 189 samples (60 HCC patients and 129 people with cirrhosis) and 185 samples (59 HCC patients and 126 people with cirrhosis), respectively^55^.

To construct training and validation datasets and sub-datasets of various sample size, random sampling and *k*-means clustering were applied. Taking MTBLS28 ESI+ as an example, 1,005 samples were divided into training dataset (400 lung cancer patients and 500 healthy individuals) and validation dataset (105 samples) by random sampling. Moreover, to generate the sub-datasets from training dataset, the *k*-means clustering^56^ was used to sample 10 sub-datasets of various sample size. In particular, the number of lung cancer patients versus that of healthy individuals were 50 *vs.* 40, 100 *vs.* 80, 150 *vs.* 120, 200 *vs.* 160, 250 *vs.* 200, 300 *vs.* 240, 350 *vs.* 280, 400 *vs.* 320, 450 *vs.* 360, and 500 *vs.* 400 for 10%, 20%, 30%, 40%, 50%, 60%, 70%, 80%, 90%, 100% of the samples in the training group, respectively.

### LC/MS based metabolomics data pre-processing

Biological variance and technical error are two key factors introducing biases to the metabolomics data. Biological variance arises from the spread of metabolic signals detected from various biological samples^57^, while technical error results from machine drift^58^. In particular, biological variances (e.g., varying concentration levels of bio-fluid, different cell sizes, varying sample measurements) are commonly encountered in metabolomics data^13^, while technical errors (e.g., a sudden drop in peak intensities or measurements on different instruments) are the major issues in large-scale metabolomics studies^58^. Apart from those above methods widely adopted to remove biological variances^22^, quality-control (QC) samples were used to significantly reduce technical errors^58^.

Moreover, sparsity is the nature of metabolomics data, which can be represented by a substantial amount of missing values (10~40%), which can affect up to 80% of all metabolic features^59^. The direct assignment of zero to the missing values could be useful for cluster analysis, but it may lead to poor performance or even malfunction if normalization method is applied^50^, especially for those methods based on the logarithm (e.g., the *Log Transformation*)^50^,^53^. Several missing value imputation methods are currently available, among which the *KNN* algorithm^60^ was reported as the most robust one for analyzing mass spectrometry based metabolomics data^60^. Therefore, the *KNN* algorithm was adopted in this work to impute the missing signals of the metabolic features.

**Figure 1:**
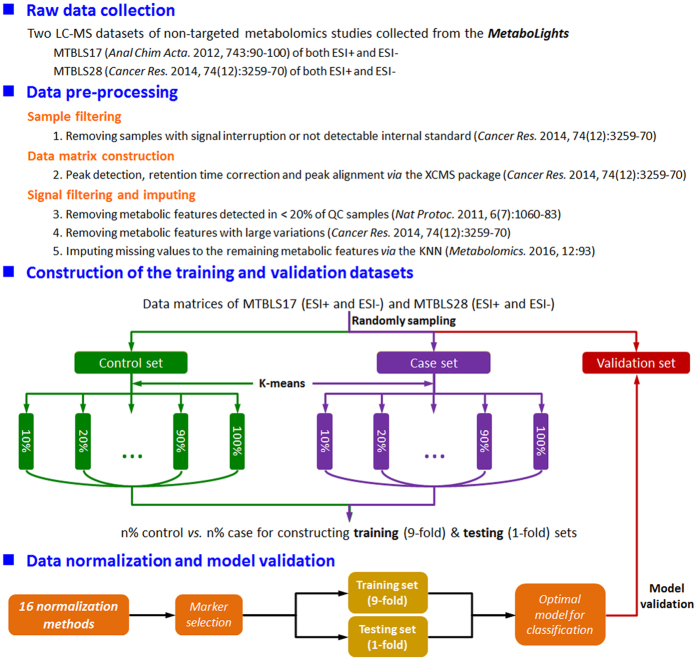
The overall research design and flowchart of this study.

In this study, a widely adopted data pre-processing procedure^54^,^60^,^61^ was applied, which included sample filtering, data matrix construction and signal filtering & imputing (Fig. 1). In particular, (1) samples with signal interruption or not detectable internal standard were removed based on Mathé’s work^54^; (2) peak detection, retention time correction and peak alignment^54^ were applied to the UHPLC/Q-TOF-MS raw data (in CDF format) using the *xcmsSet*, the *group* and the *rector* functions in the *XCMS* package^62^ with both the full width at half-maximum (fwhm) and the retention time window (bw) set as 10; (3) metabolic features detected in <20% of QC samples^61^ or with large variations^54^ were removed based on Mathé’s work, and missing signals of the remaining metabolic features were imputed by the *KNN* algorithm^60^. The detailed workflow of data pre-processing used in this study was illustrated in Fig. 1.

### Normalization methods analyzed in this study

16 methods were analyzed in this work, which include the *Auto Scaling* (unit variance scaling, UV)^38^, the *Contrast Normalization*^33^, the *Cubic Splines*^34^, the *Cyclic Locally Weighted Regression* (Cyclic Loess)^35^, the *Level Scaling*^12^, the *Linear Baseline Scaling*^25^, the *Log Transformation*^39^, the *MS Total Useful Signal* (MSTUS)^22^, the *Non-Linear Baseline Normalization* (Li-Wong)^36^, the *Pareto Scaling*^40^, the *Power Scaling*^41^, the *Probabilistic Quotient Normalization* (PQN)^37^, the *Quantile Normalization*^25^, the *Range Scaling*^42^, the *Variance Stabilization Normalization* (VSN)^43^,^44^ and the *Vast Scaling*^45^.

*Auto Scaling (unit variance scaling, UV)* is one of the simplest methods adjusting metabolic variances^21^, which scales metabolic signals based on the standard deviation of metabolomics data. This method makes all metabolites of equal importance, but analytical errors may be amplified due to dilution effects^21^. Auto scaling has been used to improve the diagnosis of bladder cancer using gas sensor arrays^63^ and to identify urinary nucleoside markers from urogenital cancer patients^64^.

*Contrast Normalization* is originated from the integration of *MA*-plots and logged *Bland-Altman* plots, which assumes the presence of non-linear biases^21^. The use of a log function in this method may impede the processing of zeros and negative numbers, which requires the conversion of non-positive numbers to an extremely small value^21^. The contrast method has been employed to reveal the role of polychlorinated biphenyls in non-alcoholic fatty liver disease by metabolomics analysis^65^.

*Cubic Splines* is one of the non-linear baseline methods assuming the existence of non-linear relationships between baseline and individual spectra^21^. Cubic splines has been adopted to reduce variability in DNA microarray experiments by normalizing all signal channels to a target array^34^. Moreover, this method has been performed to evaluate differential effects of clinical and biological variables in breast cancer patients^66^.

Similar to contrast normalization, *Cyclic Locally Weighted Regression (Cyclic Loess)* comes also from the combination of *MA*-plot and logged *Bland-Altman* plot by assuming the existence of non-linear bias^21^. However, cyclic loess is the most time-consuming one among those studied normalization methods, and the amount of time grows exponentially as the number of sample increases^67^. This method has been used to discover microRNA candidates regulating human osteosarcoma^68^.

*Level Scaling* transforms metabolic signal variation into variation relative to the average metabolic signal by scaling according to the mean signal^12^. This method is especially suitable for the circumstances when huge relative variations are of great interest (e.g., studying the stress responses, identifying relatively abundant biomarkers)^12^. Level Scaling has been used to identify urinary nucleoside markers from urogenital cancer patients^64^.

*Linear Baseline Scaling* maps each sample spectrum to the baseline based on the assumption of a constant linear relationship^21^. However, this assumption of a linear correlation among sample spectra may be oversimplified^21^. This method has been conducted to identify differential metabolomics profiles among the banana’s 5 different senescence stages^69^. Moreover, linear baseline scaling has been performed to discover the toxicity profiling of capecitabine in patients with inoperable colorectal cancer^70^.

*Log Transformation* converts skewed metabolomics data to symmetric via the non-linear transformation, which is usually used to adjust heteroscedasticity and transform metabolites’ relations from multiplication to addition^12^. In metabolomics, relations among metabolites may not always be additive, this method is thus needed to identify multiplicative relation with linear techniques^12^. This method has been used to delineate potential role of sarcosine in prostate cancer progression^71^.

*MS Total Useful Signal (MSTUS)* utilizes the total signals of metabolites that are shared by all samples by assuming that the number of increased and decreased metabolic signals is relatively equivalent^22^,^72^. However, the validity of this hypothesis is questionable since an increase in the concentration of one metabolite may not necessarily be accompanied by a decrease in that of another metabolite^72^,^73^. MSTUS has been reported as among the best choices for overcoming sample variability in urinary metabolomics^73^ and used to identify diagnostic and prognostic markers for lung cancer patients^54^.

*Non-Linear Baseline Normalization (Li-Wong)* is one of the normalization methods aiming at removing unwanted sample-to-sample variations^21^. This method is first used to analyze oligonucleotide arrays based on a multiplicative parametrization^36^,^74^, and currently adopted to improve NMR-based metabolomics analysis^21^. This method has already been successfully integrated into the *dChip*^74^.

Different from the auto scaling, *Pareto Scaling* uses the square root of the standard deviation of the data as scaling factor^40^. Therefore, comparing to the auto scaling, this method is able to reduce more significantly the weights of large fold changes in metabolite signals, but the dominant weight of extremely large fold changes may still be unchanged^21^. Pareto scaling has been performed for improving the pattern recognition for targeted^75^ and untargeted^76^ metabolomics data.

*Power Scaling* aims at correcting for the pseudo scaling and the heteroscedasticity^12^. Different from the log transformation, the method is able to handle and zero values^12^. Power scaling has been used to study the serum amino acid profiles and their variations in colorectal cancer patients^77^.

*Probabilistic Quotient Normalization (PQN)* transforms the metabolomics spectra according to an overall estimation on the most probable dilution^37^. This algorithm has been reported to be significantly robust and accurate comparing to the integral and the vector length normalizations^37^. PQN has been used to discover potential diagnostic technique for ovarian and breast cancers from urine metabolites^78^.

*Quantile Normalization* aims at achieving the same distribution of metabolic feature intensities across all samples, and the quantile-quantile plot in this method is used to visualize the distribution similarity^21^. Quantile normalization has been used to probe differential molecular profiling between pancreatic adenocarcinoma and chronic pancreatitis^79^, and currently adopted to improve NMR-based metabolomics analysis^21^.

*Range Scaling* scales the metabolic signals by the variation of biological responses^63^. A disadvantage of this method lies in a limited number (usually only 2) of values used to describe the variation unlike other scaling methods taking all measurements into account using the standard deviation, which makes this algorithm relatively sensitive to outliers^12^. Because all variation levels of the metabolites are treated equally by the range scaling, it has been used to fuse mass spectrometry-based metabolomics data^42^.

*Variance Stabilization Normalization (VSN)* is one of the non-linear methods aiming at remain variances unchanged across the whole data range^21^. The method is reported to be a preferred approach for exploratory analysis such as the principal component analysis^80^. VSN was originally developed for normalizing single and two-channel microarray data^81^, and currently used to determine metabolic profiles of liver tissue during early cancer development^82^.

As an extension of the auto scaling, *Vast Scaling* scales the metabolic signals based on the coefficient of variation^12^. Vast scaling has been used to identify prognostic factors for breast cancer patients from the magnetic resonance based metabolomics^83^.

Detailed descriptions on these methods could be found in Supplementary Note S1, and their source codes programed in this study could be found in Supplementary Note S2.

### Assessment of the normalization performance by classification algorithm

Firstly, the differential metabolic features were identified by VIP value (>1) of the *partial least squares discriminant analysis* (PLS-DA)^84^ in R package *ropls*^85^ together with p-value (<0.05) of Student *t*-test^71^. All computational assessments were conducted in R (http://www.r-project.org) version 3.2.4 running on 64-bit Mac OS X EI Capitan (v10.11.5) platform. Source codes of related programs designed in this study could be found in Supplementary Note S2.

Secondly, classification algorithm was applied to assess the performance of each normalization method based on the identified differential metabolic features. Several classification algorithms were adopted to evaluate the performance of normalization methods, which include the *Support Vector Machine* (SVM)^21^, the *k-Nearest Neighbors* (k-NN)^86^, the *Gaussian Mixture Model* (GMM)^87^, and so on. As illustrated in Fig. 1, the SVM algorithm in the R package *e1071* (http://cran.r-project.org/web/packages/e1071) was selected to assess normalization performance in this study. In the process of training the classification models, *10*-fold cross validation was used to optimize parameters, and the validation dataset was then used to assess the classification performance of the selected differential features by the receiver operating characteristic (ROC) plots generated by R package *ROCR*^88^. Source codes of the classification algorithm programed in this study could be found in Supplementary Note S2.

### Identification of the performance relationship among normalization methods

The hierarchical clustering^56^,^89^,^90^ was adopted to identify the relationship of sample size dependent performance among 16 methods. Firstly, the area under the curve values (AUCs) of a specific method among 10 sub-datasets of various sample size were used to generate a 10 dimensional vector. Secondly, hierarchical clustering was adopted to investigate the relationship among vectors, and therefore among corresponding methods. As an assessment of consistency between different distance metrics, two metrics (the *Manhattan* and the *Euclidean*) were applied:


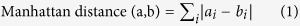



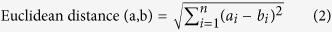


In Eq. (1) and Eq. (2), *i* refers to each AUC of method *a* and *b*. Clustering approach adopted is the Ward’s minimum variance method^91^, which is used to reduce the total within-cluster variance to the maximum extent. In this work, Ward’s minimum variance module in R package was used^92^. Source codes of the hierarchical clustering algorithm programed in this study could be found in Supplementary Note S2.

### Construction of web-based tool for evaluating performance of 16 normalization method

A web-based tool named as MetaPre for comprehensively evaluating the normalization performance of all 16 methods was constructed and hosted at http://server.idrb.cqu.edu.cn/MetaPre/. MetaPre was developed in R environment, and further extended using HTML, CSS and JavaScript. The R package *Shiny* (http://shiny.rstudio.com/) was used to construct web application (comprised of a front end and a back end). R package *DiffCorr*^93^ and *vsn* from Bioconductor Project^94^ were utilized to support background processes. MetaPre server was deployed at Apache HTTP web server v2.2.15 (http://httpd.apache.org).

## Results and Discussion

### Validation of the differential metabolic features selected based on 16 normalization methods

Supplementary Table S1 showed the number of differential metabolic features identified by PLS-DA based on 16 normalization methods. As demonstrated, the numbers of features selected based on some methods were the same as each other, while the numbers identified by some others varied significantly. SVM classifier based on those features was used in this work, the validity of these features were therefore crucial for assessing performances of 16 methods. In this study, two lines of evidence were provided for this assessment. First, a benchmark spike-in dataset from Franceschi’s work^95^ was analyzed. As shown in Supplementary Table S2, the performances on identifying spike-in compounds based on 16 methods were equivalent to that of Franceschi’s work, which indirectly reflected the reliability of strategy applied in this study. Secondly, 2 markers (creatine riboside and 561.3432) from positive and other 2 markers (cortisol sulfate and *N*-acetylneuraminic acid) from negative ionization mode were experimentally validated in Mathé’s work^54^. Supplementary Table S3 listed the number of experimentally validated markers identified by this work from the same datasets as that in Mathé’s work (MTBLS28 ESI+ and ESI−). For all methods of various sample sizes, the absolute majority (91.6%) identified all experimentally validated markers, which could server as another line of evidence for the validity of metabolic features selected by this study.

### Variation of normalization performances among 16 methods based on benchmark datasets

Table 1 demonstrated the prediction accuracy (ACC) of each method trained by 10 sub-datasets based on MTBLS28 (ESI+ and ESI−). For the training set of 900 samples from MTBLS28 ESI+, the ACC values of 11 methods fell in the range from 0.6095 (the *Level Scaling*) to 0.6952 (the *Log Transformation,* the *Power Scaling* and the *Range Scaling*). The ACC values of 4 methods (the *VSN,* the *PQN,* the *Cyclic Loess* and the *Cubic Splines*) exceeded 0.7, while that of another method (the *Contrast*) was only 0.5143. For training set of 900 samples from MTBLS28 ESI−, the ACC values of 14 methods fell in the range from 0.6095 (the *Level Scaling*) to 0.6857 (the *Cyclic Loess* and the *VSN*). The ACC value of only one method (the *Quantile*) exceeded 0.7, while that of another method (the *Contrast*) was only 0.3333. Moreover, Supplementary Table S4 showed the ACC values of each method trained by 10 sub-datasets based on MTBLS17 (ESI+ and ESI−). For the training set of 170 samples from MTBLS17, the *Contrast* method always underperformed comparing to other methods, which was similar to that of MTBLS28. However, the top-ranked normalization methods for each ionization mode of each dataset vary significantly, which is in accordance with Chawade’s conclusion that the effectiveness of a method in normalizing data relied on the nature of the analyzed data^53^. Thus, this significant variation reminded us that it is essential to take various sample size into account, if one try to compare the performance among normalization methods.

**Figure 2:**
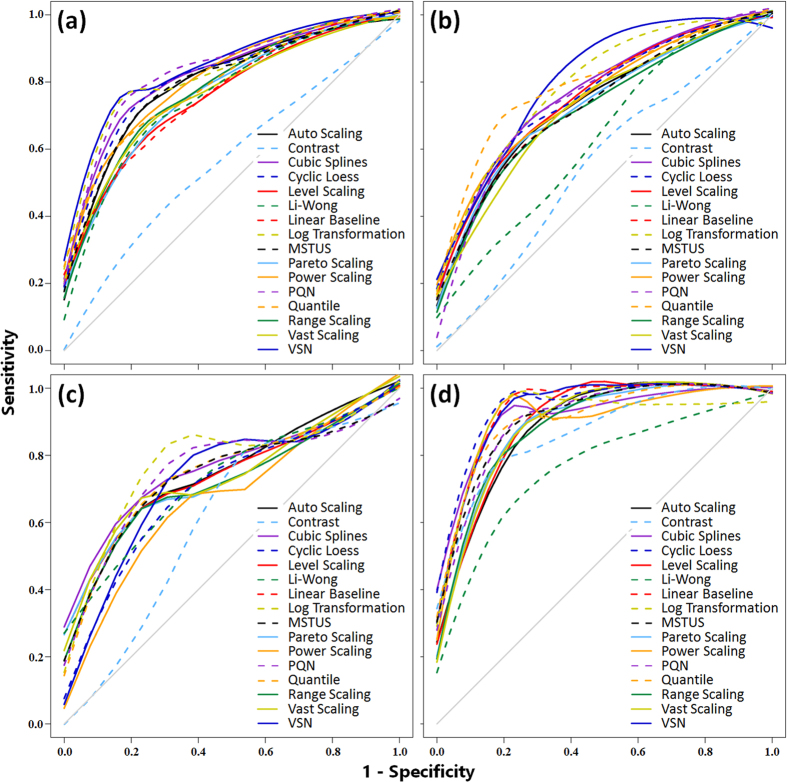
Normalization performance of 16 methods measured by receiver operating characteristic (ROC) curves based on four benchmark datasets: (**a**) MTBLS28 ESI+, (**b**) MTBLS28 ESI−, (**c**) MTBLS17 ESI+ and (**d**) MTBLS17 ESI−. The training dataset of (**a**) and (**b**) composed of 900 samples (400 lung cancer patients and 500 healthy individuals), and that of (**c**) and (**d**) consisted of 170 samples (50 HCC patients and 120 people with cirrhosis). The grey diagonal represented an invalid model with the corresponding area under the curve (AUC) value equaled to 0.5. All lines were generated by the LOESS regression.

The receiver operating characteristic (ROC) curves and the area under the curve values (ACCs) were used to illustrate the performances of 16 methods in Fig. 2 and Supplementary Table S5. Figure 2a–d illustrated ROC curves of MTBLS28 ESI+, MTBLS28 ESI−, MTBLS17 ESI+ and MTBLS17 ESI−, respectively. The training dataset of Fig. 2a and b consisted of 900 samples (400 lung cancer patients and 500 healthy individuals), and that of Fig. 2c and d consisted of 170 samples (50 HCC patients and 120 people with cirrhosis). The grey diagonal represented an invalid model with the corresponding AUC value equaled to 0.5. As illustrated in Fig. 2a–d, the *Contrast* method showed a poor normalization performance in all 4 datasets, while the *VSN* and the *Log Transformation* outperformed consistently. However, performance rank of the remaining methods fluctuated dramatically, which also requested a collective assessment of normalization performance based on various sample size.

### Categorization of 16 methods based on their normalization performances

**Figure 3:**
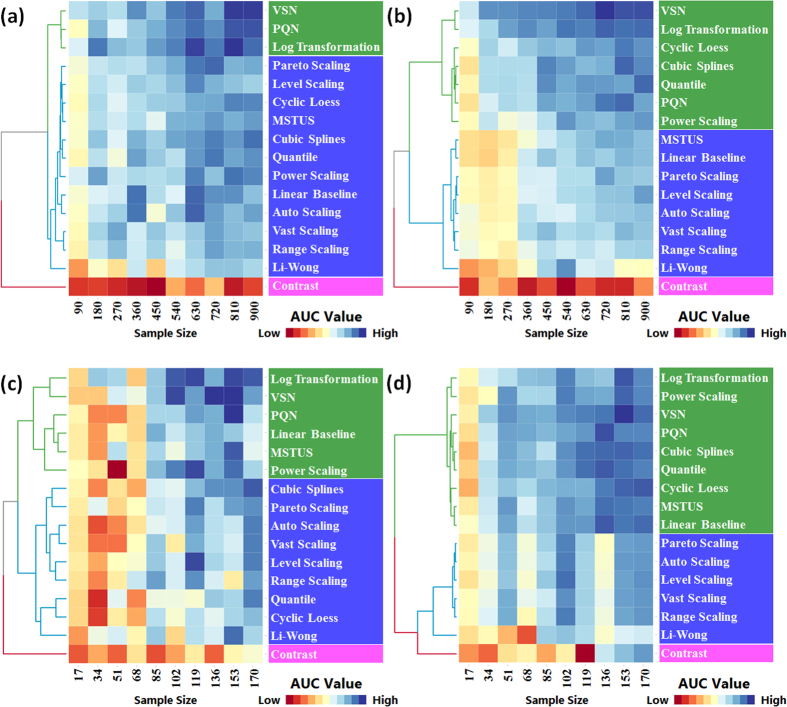
Cluster analysis of 16 normalization methods according to their AUC values (across 10 various sample sizes) calculated based on four benchmark datasets: (**a**) MTBLS28 ESI+, (**b**) MTBLS28 ESI−, (**c**) MTBLS17 ESI+ and (**d**) MTBLS17 ESI−. The data were presented in matrix format in which columns represent specific training dataset of various sample size and rows represent each normalization method. Each cell in heat map represents AUC value of a normalization method trained on one specific training sample. The cell of the highest AUC value was set as exact blue with those lower AUC values gradually fading towards red (the lowest AUC value). Hierarchical clustering analyses were conducted using *Manhattan* metric and Ward’s minimum variance algorithm.

AUCs of a specific method among 10 sub-datasets were calculated to construct a 10 dimensional vector. The resulting 16 vectors were then hierarchically clustered based on two popular distance metrics (the *Manhattan* in Fig. 3 and the *Euclidean* in Supplementary Figure S1). Cluster analysis of 16 methods was conducted based on 4 benchmark datasets: (a) MTBLS28 ESI+, (b) MTBLS28 ESI−, (c) MTBLS17 ESI+ and (d) MTBLS17 ESI−. As shown in Fig. 3a–d, 16 methods were divided by the corresponding dendrogram on the left side of each figure into three areas: top, middle and bottom areas colored by green, blue and magenta, respectively. Clearly, 3 methods (the *VSN*, the *Log Transformation* and the *PQN*) were consistently ranked into the top area of all 4 figures, while one method (the *Contrast*) always stayed in the bottom area. Therefore, 16 normalization methods could be categorized into 3 groups (A, B and C) by comprehensively considering their performances across all 4 benchmark datasets.

**Figure 4:**
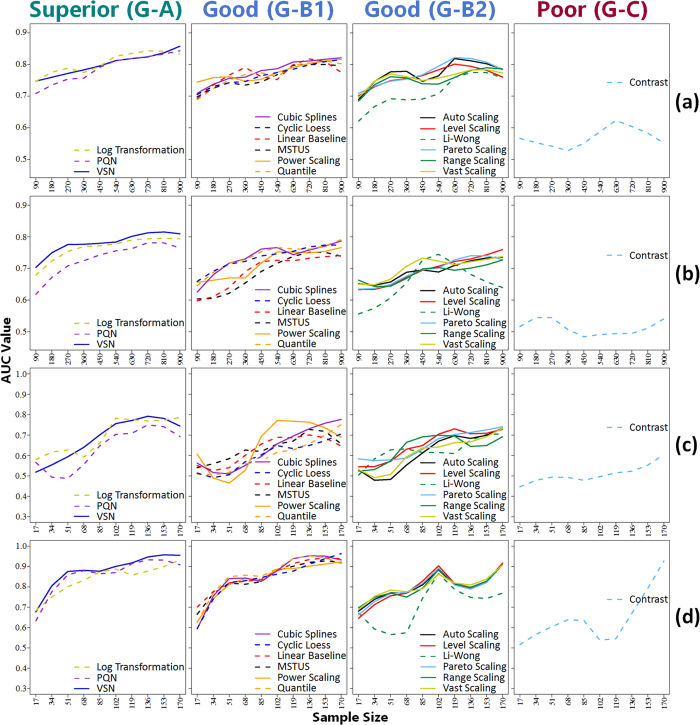
Method groups categorized according to the normalization performances across various sample sizes based on four benchmark datasets: (**a**) MTBLS28 ESI+, (**b**) MTBLS28 ESI−, (**c**) MTBLS17 ESI+ and (**d**) MTBLS17 ESI−. (G–A) superior performance group; (G-B1) good performance group including methods occasionally classified to top green area of Fig. 3; (G-B2) good performance group including methods consistently staying in middle blue area of Fig. 3; (C) poor performance group. All lines were generated by the LOESS regression.

As illustrated by Fig. 4, normalization methods in group A (the *VSN*, the *Log Transformation* and the *PQN*) demonstrated the best performance among all 16 methods, which made group A (G-A) the Superior Performance Group. The *VSN* and the *PQN* had been discovered as robust and well-performed methods in metabolomics for various dilutions of biological samples^37^,^96^. The *Log Transformation* was reported to be a powerful tool for making skewed distributions symmetric^12^, it was therefore a very suitable method for treating metabolomics data (the distribution of which was right-skewed)^23^. Moreover, some methods (e.g., the *VSN*) in G-A was also found to be the most capable one in reducing variation between technical replicates in proteomics, and consistently well-performed in identifying differential expression profiles^97^. The *Contrast* was the only one method in group C (G-C, the Poor Performance Group), the performance of which was consistently the worst across 10 sub-datasets among all 16 methods. As reported by Kohl *et al*.^21^, the *Contrast* hardly reduced bias at all and could not improve comparability among samples^21^.

Moreover, the remaining 12 methods in group B (Good Performance Group) could be further divided into G-B1 (including 6 methods occasionally classified to the top area of Fig. 3) and G-B2 (including 6 methods consistently staying in the middle area of Fig. 3). As illustrated in Fig. 4, although slightly underperformed comparing to G-A, methods in G-B1 showed good normalization performances across 10 sub-datasets of various sample size. Furthermore, the majority of the methods in G-B2 followed a similar fluctuation trends across various sample sizes, with the *Li-Wong* distinguished as an outlier. The *Li-Wong* performed the worst among other assessed methods in reducing within- and between-group variations^96^, and could hardly reduce the biases among samples at all^21^.

Similar to the *Manhattan* metric (Fig. 3), 16 methods could also be re-categorized with the *Euclidean* metric. As illustrated in Supplementary Figure S1, the categorization generated based on the *Euclidean* metric identified 3 groups with exactly the same methods in each group as that of the *Manhattan* metric, which reflected the independent nature of method categorization on different distance metrics. Moreover, in Supplementary Figure S1d, the *Li-Wong* was clustered into the bottom area (magenta) together with the *Contrast*, which again reflected its unsuitability in analyzing LC/MS based metabolomics data^21^,^96^.

### Online interactive analysis tool for normalizing LC/MS based metabolomics data

**Figure 5:**
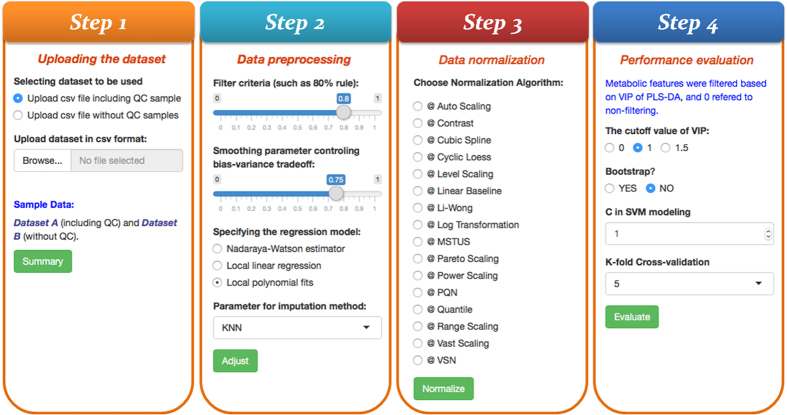
General operational procedure for using MetaPre.

With R package *Shiny* (http://shiny.rstudio.com/), an interactive web tool, named MetaPre, was developed in this study and hosted at http://server.idrb.cqu.edu.cn/MetaPre/. The MetaPre constructed to normalize LC/MS based metabolomics data could be easily accessed by modern web browsers such as Chrome, Foxfire, IE, Safari, and so on. Meanwhile, the local version of MetaPre was freely provide in this study and could also be readily downloaded from https://github.com/libcell/MetaPre in *Github*. The procedure for using online version of the MetaPre was provided in Fig. 5, which included 4 steps: (1) uploading the dataset; (2) data pre-processing; (3) data normalization; (4) performance evaluation.

*Uploading the dataset* provided the option to upload data with or without QC samples. In large-scale metabolomics study (especially the LC/MS based one), not all samples can be analyzed in the same experimental batch^61^. To cope with these difficulties, QC samples were frequently applied^58^,^61^. In the MetaPre, batch correction based on QC samples was provided, which made this tool one of the few currently available online servers^51^,^98^ offering such kind of function.

*Data pre-processing* offered the function to correct metabolic features and impute missing signals. For data with QC samples, the MetaPre firstly applied within-block signal correction^61^ to correct metabolic features. Then, multiple popular imputing algorithms were provided to fill missing signals. For data without QC samples, only the process of missing signal imputing was implemented.

*Data normalization* integrated 16 normalization methods discussed in this study to remove the unwanted biological variations. After selecting any of these methods, the normalized data matrix was displayed on the web page and a corresponding *csv* file could be downloaded directly. Moreover, two box plots used to visualizing the distributions of data before and after normalization were illustrated on the web page.

*Performance evaluation* was quantified based on AUC values of the constructed SVM models. Firstly, the differential metabolic features were identified by VIP value (>1) of PLS-DA model. Then, SVM models were constructed based on these identified differential features. After *k*-folds cross validation, ROC curve together with its AUC value were calculated and displayed on the web page.

MetaPre is valuable online tool to select suitable methods for normalizing LC/MS based metabolomics data, and is a useful complement to the currently available tools in modern metabolomics analysis.

## Conclusion

Based on the 4 datasets tested in this work, 16 methods for normalizing LC/MS based metabolomics data were categorized into three groups based on their normalization performances across various sample sizes, which included the superior (3 methods), good (12 methods) and poor (1 method) performance groups. The *VSN*, the *Log Transformation* and the *PQN* were identified as methods of the best normalization performance, while the *Contrast* consistently underperformed across all sub-datasets of different benchmark data among those 16 methods. Moreover, an interactive web tool comprehensively evaluating the performance of all 16 methods for normalizing LC/MS based metabolomics data was constructed and hosted at http://server.idrb.cqu.edu.cn/MetaPre/. In sum, this study could serve as guidance to the selection of suitable normalization methods in analyzing the LC/MS based metabolomics data.

## Additional Information

**How to cite this article**: Li, B. *et al*. Performance Evaluation and Online Realization of Data-driven Normalization Methods Used in LC/MS based Untargeted Metabolomics Analysis. *Sci. Rep.* **6**, 38881; doi: 10.1038/srep38881 (2016).

**Publisher's note:** Springer Nature remains neutral with regard to jurisdictional claims in published maps and institutional affiliations.

## Supplementary Material

Supplementary Material

## Figures and Tables

**Table 1:** Performance evaluation of 16 normalization methods across 10 sub-datasets based on the benchmark data MTBLS28 (ESI+ and ESI−).

Normalization method	MetaboLights ID (ionization mode)	Sample size of 10 various sub-datasets used in the training set									
		90	180	270	360	450	540	630	720	810	900
Auto Scaling	MTBLS28 (ESI+)	0.5905	0.6286	0.6381	0.6952	0.5810	0.6286	0.6381	0.6381	0.6476	0.6667
	MTBLS28 (ESI−)	0.5524	0.5333	0.5810	0.6190	0.6000	0.6190	0.6571	0.6476	0.6381	0.6667
Contrast	MTBLS28 (ESI+)	0.4762	0.5238	0.4095	0.4667	0.5238	0.5619	0.5429	0.5619	0.4762	0.5143
	MTBLS28 (ESI−)	0.4381	0.3429	0.4667	0.3524	0.3524	0.3714	0.4000	0.3619	0.3524	0.3333
Cubic Splines	MTBLS28 (ESI+)	0.6095	0.6095	0.6762	0.6952	0.6095	0.7143	0.6476	0.7048	0.6762	0.7048
	MTBLS28 (ESI−)	0.6667	0.6095	0.6381	0.6000	0.6476	0.6952	0.6952	0.6762	0.7238	0.6667
Cyclic Loess	MTBLS28 (ESI+)	0.6000	0.6190	0.6667	0.6381	0.6476	0.6476	0.6381	0.6762	0.7524	0.7238
	MTBLS28 (ESI−)	0.5905	0.6667	0.6571	0.6190	0.6476	0.6667	0.6857	0.6762	0.6857	0.6857
Level Scaling	MTBLS28 (ESI+)	0.5905	0.6190	0.6381	0.6476	0.6286	0.6476	0.6286	0.6381	0.6286	0.6095
	MTBLS28 (ESI−)	0.5714	0.5619	0.6095	0.6190	0.6000	0.6095	0.6667	0.6476	0.6381	0.6095
Li-Wong	MTBLS28 (ESI+)	0.5524	0.6095	0.5524	0.6000	0.5619	0.5714	0.6190	0.6286	0.6667	0.6762
	MTBLS28 (ESI−)	0.5048	0.6286	0.5429	0.6571	0.6476	0.6762	0.6381	0.5905	0.6476	0.6286
Linear Baseline	MTBLS28 (ESI+)	0.6095	0.6095	0.6476	0.7238	0.6095	0.6286	0.6667	0.6762	0.6952	0.6286
	MTBLS28 (ESI−)	0.5619	0.5619	0.5905	0.6095	0.6190	0.6000	0.6762	0.6571	0.6286	0.6476
Log Transformation	MTBLS28 (ESI+)	0.6476	0.6857	0.6952	0.6476	0.7048	0.6667	0.6857	0.6952	0.6762	0.6952
	MTBLS28 (ESI−)	0.6095	0.6286	0.6190	0.6762	0.6095	0.6571	0.6857	0.6190	0.6952	0.6476
MSTUS	MTBLS28 (ESI+)	0.5905	0.6095	0.6286	0.6476	0.6000	0.6571	0.6667	0.6762	0.6667	0.6762
	MTBLS28 (ESI−)	0.5619	0.5714	0.5619	0.6000	0.6095	0.6286	0.6762	0.6762	0.6667	0.6476
Pareto Scaling	MTBLS28 (ESI+)	0.6190	0.6571	0.6190	0.5905	0.6476	0.6667	0.6476	0.6952	0.6381	0.6667
	MTBLS28 (ESI−)	0.5714	0.5619	0.5905	0.6095	0.6000	0.6190	0.6667	0.6381	0.6381	0.6476
Power Scaling	MTBLS28 (ESI+)	0.6000	0.6571	0.5905	0.6190	0.6000	0.6286	0.6952	0.6476	0.7048	0.6952
	MTBLS28 (ESI−)	0.5810	0.6381	0.5905	0.5714	0.5810	0.6095	0.6476	0.6095	0.6286	0.6476
PQN	MTBLS28 (ESI+)	0.5619	0.6381	0.6476	0.6286	0.6381	0.6667	0.6762	0.6952	0.7524	0.7333
	MTBLS28 (ESI−)	0.6000	0.6095	0.6476	0.6381	0.6571	0.6857	0.6667	0.7238	0.7429	0.6667
Quantile	MTBLS28 (ESI+)	0.6095	0.6190	0.6476	0.6952	0.6095	0.6476	0.6762	0.7143	0.7048	0.6857
	MTBLS28 (ESI−)	0.6667	0.6190	0.6381	0.6095	0.6286	0.6667	0.6857	0.6571	0.6381	0.7238
Range Scaling	MTBLS28 (ESI+)	0.5714	0.6190	0.6190	0.5905	0.6381	0.6000	0.6190	0.6476	0.6381	0.6952
	MTBLS28 (ESI−)	0.5810	0.5429	0.5714	0.5905	0.6000	0.6095	0.6190	0.6476	0.6667	0.6571
Vast Scaling	MTBLS28 (ESI+)	0.5524	0.6190	0.6286	0.5905	0.6381	0.5905	0.6286	0.6571	0.6476	0.6381
	MTBLS28 (ESI−)	0.5333	0.5714	0.6095	0.6095	0.6095	0.5810	0.6571	0.6190	0.6667	0.6190
VSN	MTBLS28 (ESI+)	0.6381	0.6381	0.6286	0.7048	0.6476	0.7048	0.7048	0.6571	0.7524	0.7429
	MTBLS28 (ESI−)	0.6571	0.6762	0.6476	0.6762	0.6476	0.6667	0.6762	0.7048	0.6857	0.6857

The performance was evaluated by the prediction accuracies (ACCs) on the validation set. The ACC equals to (true positive + true negative)/(true positive + false positive + true negative + false negative).
