# Supplementary material for: Performance Evaluation and Online Realization of Data-driven Normalization Methods Used in LC/MS based Untargeted Metabolomics Analysis
Source: Sci Rep. 2016 Dec 13;6:38881. doi: 10.1038/srep38881 (PMC5153651; doi:10.1038/srep38881)
Supplement: Supplementary Material [file srep38881-s1.pdf]

Supplementary Information for:

Performance Evaluation and Online Realization of Data-driven Normalization Methods Used in LC/MS based Untargeted Metabolomics Analysis

Bo Li[1,+], Jing Tang[1,+], Qingxia Yang[1,+], Xuejiao Cui[1], Shuang Li[1], Sijie Chen[2], Quanxing Cao[1], Weiwei Xue[1], Na Chen[1] and Feng Zhu[1,*]

[1] Innovative Drug Research and Bioinformatics Group, Innovative Drug Research Centre and School of Pharmaceutical Sciences, Chongqing University, Chongqing 401331, China

[2] College of Mathematics and Statistics, Chongqing University, Chongqing 401331, China

[+] These authors contribute equally

***** Correspondence: Feng Zhu (zhufeng@cqu.edu.cn)

Supplementary FIGURES

Figure S1. Cluster analysis of 16 normalization methods according to their AUC values (across 10 various sample sizes) calculated based on four benchmark datasets: (a) MTBLS28 ESI+, (b) MTBLS28 ESI-, (c) MTBLS17 ESI+ and (d) MTBLS17 ESI-. The data were presented in matrix format in which columns represent specific training dataset of various sample size and rows represent each normalization method. Each cell in heat map represents AUC value of a normalization method trained on one specific training sample. The cell of the highest AUC value was set as exact blue with those lower AUC values gradually fading towards red (the lowest AUC value). Hierarchical clustering analyses were conducted using *Euclidean* metric and Ward's minimum variance algorithm.

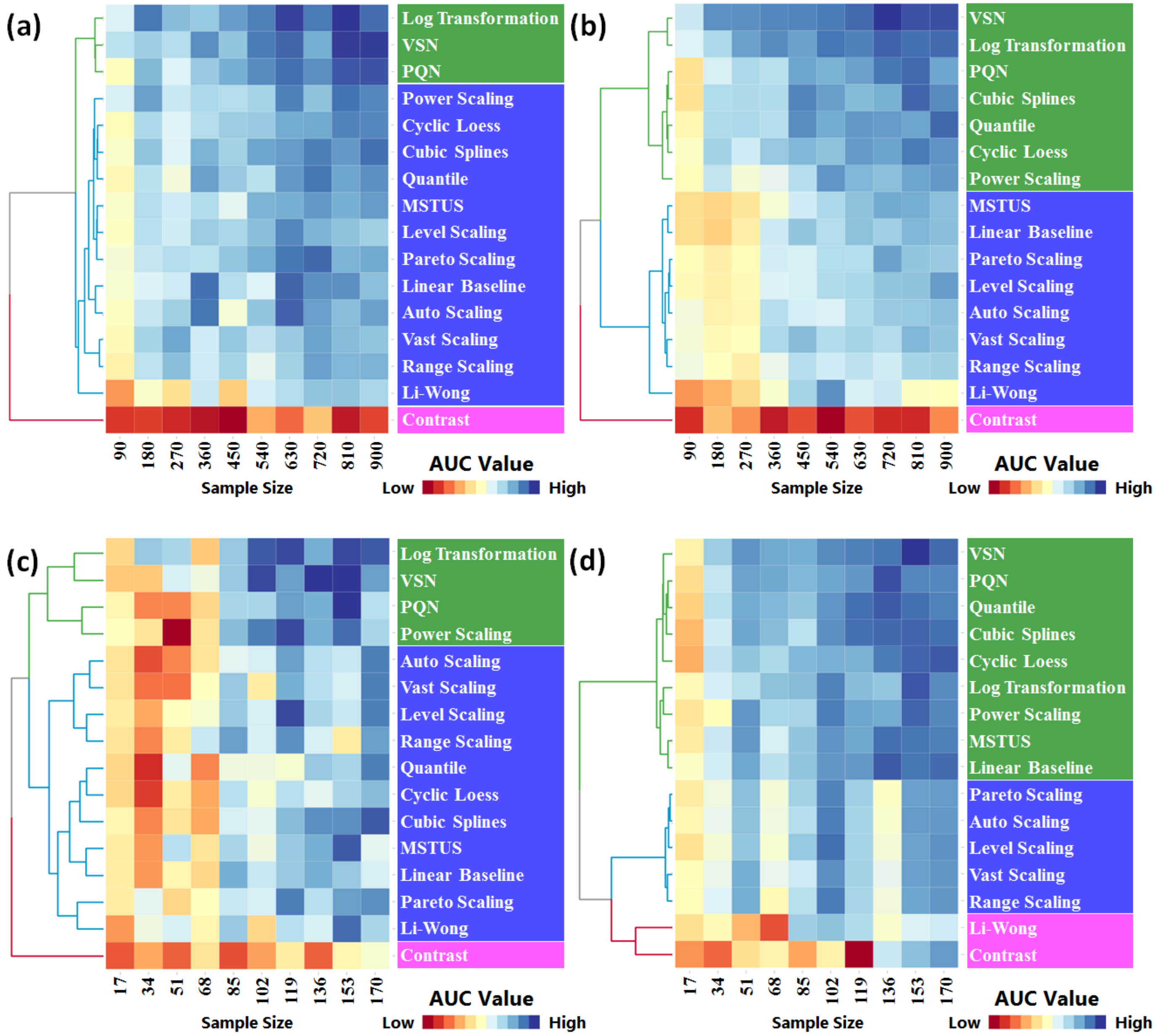

Supplementary TABLES

Table S1. Numbers of selected differential features of each normalization method across 10 sub-datasets based on the benchmark data MTBLS28 (ESI+ and ESI-) and MTBLS17 (ESI+ and ESI-). The differential metabolic features were identified by the VIP value (> 1) of S-plots and the p-value (< 0.05) of the Student *t*-test.

Normalization method	MetaboLights ID (ionization mode)	Percentage of data used in the training set for each sub-dataset									
		10%	20%	30%	40%	50%	60%	70%	80%	90%	100%
Auto Scaling	MTBLS28 (ESI+)	146	304	424	408	471	490	494	500	504	523
	MTBLS28 (ESI-)	84	239	330	294	320	329	353	367	385	382
	MTBLS17 (ESI+)	23	23	37	40	46	122	109	89	155	267
	MTBLS17 (ESI-)	42	58	21	14	22	42	58	68	64	79
Contrast	MTBLS28 (ESI+)	48	34	32	45	81	52	72	94	69	139
	MTBLS28 (ESI-)	60	2	64	16	43	37	30	19	31	34
	MTBLS17 (ESI+)	35	5	4	3	5	14	16	12	21	21
	MTBLS17 (ESI-)	26	4	4	7	4	5	10	13	21	24
Cubic Splines	MTBLS28 (ESI+)	168	333	434	449	501	522	499	522	515	540
	MTBLS28 (ESI-)	120	206	319	321	348	356	370	395	388	386
	MTBLS17 (ESI+)	30	51	31	59	62	142	118	127	200	215
	MTBLS17 (ESI-)	68	72	62	66	59	74	86	61	112	120
Cyclic Loess	MTBLS28 (ESI+)	149	310	413	424	466	496	476	503	501	517
	MTBLS28 (ESI-)	108	182	300	306	337	352	353	398	402	382
	MTBLS17 (ESI+)	32	64	45	66	77	144	142	144	213	245
	MTBLS17 (ESI-)	59	66	42	32	31	54	47	63	94	87
Level Scaling	MTBLS28 (ESI+)	146	304	424	408	471	490	494	500	504	523
	MTBLS28 (ESI-)	84	239	330	294	320	329	353	367	385	382
	MTBLS17 (ESI+)	23	23	37	40	46	122	109	89	155	267
	MTBLS17 (ESI-)	42	58	21	14	22	42	58	68	64	79
Li-Wong	MTBLS28 (ESI+)	157	258	355	314	383	420	416	477	399	489
	MTBLS28 (ESI-)	58	154	284	144	189	203	227	248	241	244
	MTBLS17 (ESI+)	17	24	38	28	55	108	90	38	95	215
	MTBLS17 (ESI-)	27	25	2	3	4	18	21	40	44	49
Linear Baseline	MTBLS28 (ESI+)	133	298	423	422	503	505	511	521	510	543
	MTBLS28 (ESI-)	79	203	293	288	288	326	355	367	377	356
	MTBLS17 (ESI+)	32	35	14	41	52	111	103	110	187	199
	MTBLS17 (ESI-)	41	67	46	43	31	62	67	51	102	100

Log Transformation	MTBLS28 (ESI+)	227	356	451	445	499	534	533	548	517	550
	MTBLS28 (ESI-)	137	291	358	316	333	351	332	366	391	376
	MTBLS17 (ESI+)	59	8	4	17	27	32	55	60	79	101
	MTBLS17 (ESI-)	91	101	61	54	63	95	90	96	127	136
MSTUS	MTBLS28 (ESI+)	133	298	423	422	503	505	511	521	510	543
	MTBLS28 (ESI-)	79	203	293	288	288	326	355	367	377	356
	MTBLS17 (ESI+)	32	35	14	41	52	111	103	110	187	199
	MTBLS17 (ESI-)	41	67	46	43	31	62	67	51	102	100
Pareto Scaling	MTBLS28 (ESI+)	146	304	424	408	471	490	494	500	504	523
	MTBLS28 (ESI-)	84	239	330	294	320	329	353	367	385	382
	MTBLS17 (ESI+)	23	23	37	40	46	122	109	89	155	267
	MTBLS17 (ESI-)	42	58	21	14	22	42	58	68	64	79
Power Scaling	MTBLS28 (ESI+)	196	375	581	563	610	635	630	604	617	608
	MTBLS28 (ESI-)	118	335	484	457	458	451	464	458	476	469
	MTBLS17 (ESI+)	42	21	17	37	41	85	89	79	147	215
	MTBLS17 (ESI-)	67	91	29	21	37	71	87	130	135	154
PQN	MTBLS28 (ESI+)	154	311	472	479	550	559	558	547	521	529
	MTBLS28 (ESI-)	112	196	338	323	373	391	390	425	412	393
	MTBLS17 (ESI+)	27	54	23	63	61	112	125	123	191	210
	MTBLS17 (ESI-)	48	71	43	29	39	71	58	56	109	108
Quantile	MTBLS28 (ESI+)	158	315	420	442	484	510	496	506	501	533
	MTBLS28 (ESI-)	120	198	320	315	344	353	359	389	390	385
	MTBLS17 (ESI+)	33	50	31	59	65	146	124	127	200	217
	MTBLS17 (ESI-)	68	68	55	60	63	75	76	63	115	115
Range Scaling	MTBLS28 (ESI+)	146	304	424	408	471	490	494	500	504	523
	MTBLS28 (ESI-)	84	239	330	294	320	329	353	367	385	382
	MTBLS17 (ESI+)	23	23	37	40	46	122	109	89	155	267
	MTBLS17 (ESI-)	42	58	21	14	22	42	58	68	64	79
Vast Scaling	MTBLS28 (ESI+)	146	304	424	408	471	490	494	500	504	523
	MTBLS28 (ESI-)	84	230	323	267	288	320	335	352	371	366
	MTBLS17 (ESI+)	23	23	37	38	41	121	108	86	148	236
	MTBLS17 (ESI-)	42	55	21	14	22	40	49	73	60	74
VSN	MTBLS28 (ESI+)	178	362	464	504	521	544	529	552	530	561
	MTBLS28 (ESI-)	156	251	365	338	391	383	404	413	427	422
	MTBLS17 (ESI+)	55	59	45	58	76	143	122	158	234	248
	MTBLS17 (ESI-)	50	74	62	51	58	81	76	65	132	129

Table S2. The number of identified features of spiked-in compound with respect to the total number of selected metabolic features from the benchmark dataset MTBLS59 (ESI+) based on each normalization method. The differential features were identified by the VIP value (> 1) of S-plots in this study, which is the same as the method used in Franceschi's work (*J. Chemometrics*. 2012, 26:16-24). Gr. 1, Gr. 2 and Gr. 3 here refer to three groups with spiked-in compounds of different concentrations.

		Number of identified spiked-in compounds / number of selected features		
		Gr. 1	Gr. 2	Gr. 3
Franceschi's work		18 / 479	16 / 503	17 / 450
This study	Auto Scaling	18 / 479	16 / 503	17 / 450
	Contrast	14 / 250	6 / 244	9 / 390
	Cubic Splines	18 / 498	16 / 509	17 / 462
	Cyclic Loess	18 / 494	16 / 504	17 / 453
	Level Scaling	18 / 479	16 / 503	17 / 450
	Li-Wong	18 / 464	16 / 456	18 / 420
	Linear Baseline	18 / 500	16 / 510	17 / 448
	Log Transformation	18 / 481	17 / 502	17 / 445
	MSTUS	18 / 500	16 / 510	17 / 448
	Pareto Scaling	18 / 479	16 / 503	17 / 450
	Power Scaling	18 / 480	16 / 498	17 / 441
	PQN	18 / 494	16 / 511	17 / 461
	Quantile	11 / 500	6 / 510	7 / 465
	Range Scaling	18 / 479	16 / 503	17 / 450
	Vast Scaling	18 / 479	16 / 503	17 / 450
	VSN	18 / 489	17 / 504	17 / 455

Table S3. The number of experimentally validated markers identified from the benchmark data MTBLS28 (ESI+ and ESI-) based on each normalization method across 10 sub-datasets. The differential features were identified by the VIP value (> 1) of S-plots and the p-value (< 0.05) of the Student *t*-test in this study. In Mathé's work (*Cancer Res.* 2014, 74(12):3259-70), 2 markers (creatine riboside and 561.3432) identified from ESI+ and other 2 markers (cortisol sulfate and *N*-acetylneuraminic acid) discovered from ESI- were experimentally validated.

Normalization Methods	Ion mode	Sample size of 10 various sub-datasets used in the training set									
		90	180	270	360	450	540	630	720	810	900
Auto Scaling	ESI+	2	2	2	2	2	2	2	2	2	2
	ESI-	1	1	2	2	2	2	2	2	2	2
Contrast	ESI+	2	2	2	2	2	2	2	2	2	2
	ESI-	1	1	2	2	2	2	2	2	2	2
Cubic Splines	ESI+	2	2	2	2	2	2	2	2	2	2
	ESI-	1	2	2	2	2	2	2	2	2	2
Cyclic Loess	ESI+	2	2	2	2	2	2	2	2	2	2
	ESI-	1	1	2	2	2	2	2	2	2	2
Level Scaling	ESI+	2	2	2	2	2	2	2	2	2	2
	ESI-	1	1	2	2	2	2	2	2	2	2
Li-Wong	ESI+	2	2	2	2	2	2	2	2	2	2
	ESI-	0	1	1	2	2	2	2	2	1	2
Linear Baseline	ESI+	2	2	2	2	2	2	2	2	2	2
	ESI-	1	1	2	2	2	2	2	2	2	2
Log Transformation	ESI+	2	2	2	2	2	2	2	2	2	2
	ESI-	2	2	2	2	2	2	2	2	2	2
MSTUS	ESI+	2	2	2	2	2	2	2	2	2	2
	ESI-	1	1	2	2	2	2	2	2	2	2
Pareto Scaling	ESI+	2	2	2	2	2	2	2	2	2	2
	ESI-	1	1	2	2	2	2	2	2	2	2
Power Scaling	ESI+	2	2	2	2	2	2	2	2	2	2
	ESI-	2	2	2	2	2	2	2	2	2	2
PQN	ESI+	2	2	2	2	2	2	2	2	2	2
	ESI-	1	1	2	2	2	2	2	2	2	2
Quantile	ESI+	2	2	2	2	2	2	2	2	2	2
	ESI-	1	1	2	2	2	2	2	2	2	2
Range Scaling	ESI+	2	2	2	2	2	2	2	2	2	2
	ESI-	1	1	2	2	2	2	2	2	2	2
Vast Scaling	ESI+	2	2	2	2	2	2	2	2	2	2
	ESI-	1	1	2	2	2	2	2	2	2	2
VSN	ESI+	2	2	2	2	2	2	2	2	2	2
	ESI-	2	2	2	2	2	2	2	2	2	2

Table S4. Performance evaluation of 16 normalization methods across 10 sub-datasets based on the benchmark data MTBLS17 (ESI+ and ESI-). The performance was evaluated by the prediction accuracies (ACCs) on the validation set. The ACC equals to (true positive + true negative) / (true positive + false positive + true negative + false negative).

Normalization method	MetaboLights ID (ionization mode)	Sample size of 10 various sub-datasets used in the training set									
		17	34	51	68	85	102	119	136	153	170
Auto Scaling	MTBLS17 (ESI+)	0.7895	0.6842	0.6842	0.7368	0.7895	0.7895	0.7895	0.7368	0.7368	0.7895
	MTBLS17 (ESI-)	0.5778	0.5778	0.6667	0.6889	0.6889	0.7556	0.6667	0.6889	0.6889	0.6889
Contrast	MTBLS17 (ESI+)	0.6316	0.6842	0.6842	0.6842	0.6842	0.6842	0.6842	0.6842	0.6842	0.6842
	MTBLS17 (ESI-)	0.5778	0.5778	0.6444	0.6444	0.5778	0.5778	0.5778	0.6000	0.6889	0.5778
Cubic Splines	MTBLS17 (ESI+)	0.6316	0.6316	0.6842	0.6842	0.7368	0.7368	0.7368	0.7368	0.7368	0.8421
	MTBLS17 (ESI-)	0.6000	0.6889	0.8222	0.5778	0.6889	0.8444	0.8222	0.8000	0.8000	0.8000
Cyclic Loess	MTBLS17 (ESI+)	0.6316	0.6316	0.6842	0.6842	0.6842	0.6842	0.6316	0.6316	0.6316	0.7368
	MTBLS17 (ESI-)	0.5778	0.6444	0.7778	0.6889	0.7778	0.8000	0.7556	0.7556	0.7111	0.7556
Level Scaling	MTBLS17 (ESI+)	0.7895	0.6842	0.7368	0.7368	0.7895	0.7895	0.7895	0.7895	0.7368	0.7895
	MTBLS17 (ESI-)	0.5333	0.6444	0.7111	0.6889	0.7111	0.7111	0.6889	0.6667	0.6889	0.6889
Li-Wong	MTBLS17 (ESI+)	0.7895	0.6842	0.7368	0.7368	0.7895	0.7895	0.7895	0.7895	0.7368	0.7895
	MTBLS17 (ESI-)	0.5333	0.6444	0.7111	0.6889	0.7111	0.7111	0.6889	0.6667	0.6889	0.6889
Linear Baseline	MTBLS17 (ESI+)	0.7368	0.6842	0.7368	0.6842	0.7895	0.7895	0.7895	0.7895	0.7895	0.7895
	MTBLS17 (ESI-)	0.5556	0.5778	0.6444	0.5778	0.7111	0.7111	0.6667	0.6000	0.6667	0.6667
Log Transformation	MTBLS17 (ESI+)	0.6316	0.6316	0.6842	0.6316	0.7895	0.7368	0.7368	0.6316	0.7368	0.6842
	MTBLS17 (ESI-)	0.6222	0.7111	0.7778	0.7333	0.8000	0.8000	0.8222	0.8889	0.7111	0.8000
MSTUS	MTBLS17 (ESI+)	0.6316	0.6842	0.6842	0.6842	0.8947	0.7895	0.8421	0.7368	0.8421	0.7895
	MTBLS17 (ESI-)	0.6444	0.6667	0.6889	0.6000	0.7556	0.8222	0.8000	0.6889	0.7778	0.6444
Pareto Scaling	MTBLS17 (ESI+)	0.6316	0.6316	0.7368	0.6316	0.7895	0.7368	0.7368	0.6316	0.7895	0.7368
	MTBLS17 (ESI-)	0.6222	0.7111	0.8000	0.7333	0.7556	0.8000	0.8222	0.8222	0.6889	0.7333
Power Scaling	MTBLS17 (ESI+)	0.6316	0.6842	0.6842	0.7895	0.7895	0.7895	0.7895	0.7368	0.7895	0.7895
	MTBLS17 (ESI-)	0.5333	0.5778	0.6667	0.6889	0.7111	0.7778	0.7111	0.6667	0.6889	0.6889
PQN	MTBLS17 (ESI+)	0.6842	0.7368	0.5789	0.6842	0.7895	0.7895	0.8421	0.6842	0.8421	0.7368
	MTBLS17 (ESI-)	0.5556	0.6667	0.7111	0.6889	0.7111	0.7556	0.7556	0.6667	0.6667	0.7333

Quantile	MTBLS17 (ESI+)	0.5789	0.5263	0.6316	0.6842	0.6842	0.7368	0.7895	0.6842	0.7895	0.6842
	MTBLS17 (ESI-)	0.6222	0.7333	0.7556	0.6889	0.7333	0.8444	0.8222	0.8667	0.7333	0.7111
Range Scaling	MTBLS17 (ESI+)	0.6316	0.6316	0.6842	0.6842	0.7368	0.6842	0.6842	0.5789	0.6842	0.7368
	MTBLS17 (ESI-)	0.6000	0.7333	0.8222	0.5778	0.7333	0.8667	0.7556	0.7556	0.8000	0.7778
Vast Scaling	MTBLS17 (ESI+)	0.7368	0.7895	0.6842	0.6842	0.7895	0.7895	0.7895	0.6842	0.6842	0.7895
	MTBLS17 (ESI-)	0.5778	0.6222	0.7333	0.6667	0.7111	0.7333	0.6889	0.6889	0.7111	0.6889
VSN	MTBLS17 (ESI+)	0.7895	0.7368	0.6842	0.7368	0.7895	0.7895	0.7368	0.7895	0.6842	0.7368
	MTBLS17 (ESI-)	0.5778	0.6222	0.7556	0.6444	0.7111	0.7778	0.7111	0.6667	0.7111	0.6889

Table S5. The area under the curve values (AUC) of each normalization method across 10 sub-datasets based on the benchmark data MTBLS28 (ESI+ and ESI-) and MTBLS17 (ESI+ and ESI-).

Normalization method	MetaboLights ID (ionization mode)	Percentage of data used in the training set for each sub-dataset									
		10%	20%	30%	40%	50%	60%	70%	80%	90%	100%
Auto Scaling	MTBLS28 (ESI+)	0.6928	0.7407	0.7645	0.8237	0.7029	0.7721	0.8341	0.7991	0.7786	0.7975
	MTBLS28 (ESI-)	0.6618	0.6296	0.6353	0.7093	0.6876	0.6836	0.7134	0.7283	0.7303	0.7367
	MTBLS17 (ESI+)	0.5513	0.4359	0.4744	0.5513	0.6282	0.6410	0.7308	0.6667	0.6538	0.7564
	MTBLS17 (ESI-)	0.6721	0.7368	0.8441	0.7267	0.7996	0.9211	0.8178	0.7045	0.8907	0.8927
Contrast	MTBLS28 (ESI+)	0.5602	0.5644	0.5499	0.5362	0.5240	0.6232	0.5880	0.6389	0.5401	0.5670
	MTBLS28 (ESI-)	0.4968	0.5849	0.5545	0.4803	0.5141	0.4622	0.5167	0.4915	0.4911	0.5503
	MTBLS17 (ESI+)	0.4423	0.5000	0.4487	0.5513	0.4359	0.4936	0.5641	0.4487	0.5769	0.6026
	MTBLS17 (ESI-)	0.5425	0.5000	0.6336	0.6680	0.5628	0.6640	0.3887	0.7773	0.8462	0.8897
Cubic Splines	MTBLS28 (ESI+)	0.6965	0.7733	0.7230	0.7862	0.7633	0.7955	0.8015	0.8180	0.8031	0.8261
	MTBLS28 (ESI-)	0.6107	0.7126	0.7146	0.7130	0.7788	0.7621	0.7419	0.7496	0.7975	0.7754
	MTBLS17 (ESI+)	0.5769	0.4744	0.5513	0.5000	0.6410	0.6282	0.7051	0.7436	0.7436	0.7821
	MTBLS17 (ESI-)	0.5830	0.7672	0.8785	0.8502	0.7915	0.9028	0.9372	0.9433	0.9474	0.9352
Cyclic Loess	MTBLS28 (ESI+)	0.6860	0.7560	0.7234	0.7524	0.7665	0.7689	0.7911	0.7931	0.8156	0.8132
	MTBLS28 (ESI-)	0.6477	0.7224	0.6904	0.7301	0.7436	0.7359	0.7653	0.7581	0.7858	0.7689
	MTBLS17 (ESI+)	0.5385	0.4231	0.5641	0.5000	0.6667	0.6026	0.6667	0.6282	0.6795	0.7051
	MTBLS17 (ESI-)	0.5709	0.7874	0.8381	0.8097	0.8563	0.8664	0.8644	0.9231	0.9474	0.9575
Level Scaling	MTBLS28 (ESI+)	0.6934	0.7496	0.7331	0.7673	0.7589	0.7774	0.8136	0.7854	0.7746	0.7645
	MTBLS28 (ESI-)	0.6381	0.6264	0.6365	0.6824	0.6856	0.7130	0.7174	0.7329	0.7351	0.7629
	MTBLS17 (ESI+)	0.5641	0.5000	0.5897	0.6026	0.6923	0.6410	0.7949	0.6795	0.6538	0.7564
	MTBLS17 (ESI-)	0.6356	0.7126	0.8360	0.7085	0.8340	0.9372	0.8178	0.7146	0.8826	0.9008
Li-Wong	MTBLS28 (ESI+)	0.6115	0.6961	0.6590	0.7379	0.6445	0.7309	0.7524	0.7729	0.7729	0.7589
	MTBLS28 (ESI-)	0.5564	0.5753	0.6039	0.6514	0.7204	0.7717	0.6854	0.6940	0.6467	0.6433
	MTBLS17 (ESI+)	0.4872	0.6154	0.6410	0.5769	0.6923	0.5385	0.6667	0.6410	0.7692	0.6795
	MTBLS17 (ESI-)	0.6296	0.6802	0.5810	0.4798	0.8259	0.8421	0.8057	0.7024	0.7551	0.7692
Linear Baseline	MTBLS28 (ESI+)	0.7007	0.7258	0.7351	0.8257	0.7520	0.7246	0.8323	0.8076	0.8084	0.7762
	MTBLS28 (ESI-)	0.6067	0.5954	0.6246	0.6930	0.7323	0.7107	0.7363	0.7242	0.7444	0.7383
	MTBLS17 (ESI+)	0.5641	0.4872	0.5769	0.5385	0.7179	0.6538	0.6923	0.7179	0.6923	0.6410
	MTBLS17 (ESI-)	0.6984	0.7652	0.8775	0.7935	0.8381	0.8947	0.8927	0.9575	0.9291	0.9413
Log Transformation	MTBLS28 (ESI+)	0.7264	0.8217	0.7790	0.7705	0.8007	0.8176	0.8510	0.8209	0.8575	0.8281
	MTBLS28 (ESI-)	0.6810	0.7166	0.7609	0.7746	0.7601	0.7882	0.7810	0.7995	0.7915	0.7947
	MTBLS17 (ESI+)	0.5385	0.6923	0.6795	0.5256	0.6923	0.7821	0.7949	0.7179	0.7949	0.7821

	MTBLS17 (ESI-)	0.6781	0.7632	0.7976	0.8441	0.8502	0.9170	0.8502	0.8360	0.9615	0.9089
MSTUS	MTBLS28 (ESI+)	0.6963	0.7520	0.7337	0.7536	0.7202	0.7862	0.7882	0.8031	0.7882	0.8007
	MTBLS28 (ESI-)	0.6067	0.6002	0.6151	0.6566	0.6904	0.7166	0.7359	0.7540	0.7516	0.7379
	MTBLS17 (ESI+)	0.5641	0.4872	0.6667	0.5513	0.6795	0.6154	0.6923	0.7179	0.7821	0.6282
	MTBLS17 (ESI-)	0.6518	0.7753	0.8927	0.7591	0.8381	0.8988	0.8765	0.9372	0.9291	0.9190
Pareto Scaling	MTBLS28 (ESI+)	0.7033	0.7415	0.7528	0.7468	0.7717	0.7899	0.8221	0.8309	0.7858	0.7919
	MTBLS28 (ESI-)	0.6409	0.6256	0.6397	0.6884	0.6860	0.7114	0.7114	0.7605	0.7323	0.7287
	MTBLS17 (ESI+)	0.5641	0.6282	0.5385	0.5897	0.6538	0.6410	0.7564	0.6667	0.7308	0.7436
	MTBLS17 (ESI-)	0.6538	0.7247	0.8462	0.7166	0.8219	0.9170	0.8239	0.6964	0.8846	0.8907
Power Scaling	MTBLS28 (ESI+)	0.7287	0.7975	0.7395	0.7572	0.7528	0.7609	0.8166	0.7750	0.8245	0.8132
	MTBLS28 (ESI-)	0.6401	0.7009	0.6578	0.6727	0.7089	0.7677	0.7452	0.7379	0.7576	0.7657
	MTBLS17 (ESI+)	0.5897	0.5513	0.3718	0.5513	0.7051	0.7564	0.7949	0.7179	0.7692	0.6795
	MTBLS17 (ESI-)	0.6417	0.6883	0.8968	0.8138	0.8158	0.9211	0.8785	0.8725	0.9474	0.9109
PQN	MTBLS28 (ESI+)	0.6900	0.7818	0.7246	0.7689	0.7882	0.8108	0.8269	0.8092	0.8406	0.8414
	MTBLS28 (ESI-)	0.6107	0.6864	0.7162	0.7081	0.7579	0.7488	0.7605	0.7866	0.7951	0.7572
	MTBLS17 (ESI+)	0.5769	0.4744	0.4744	0.5385	0.6795	0.6795	0.7308	0.7179	0.8077	0.6667
	MTBLS17 (ESI-)	0.6275	0.7814	0.8826	0.8765	0.8563	0.8785	0.8947	0.9676	0.9130	0.9130
Quantile	MTBLS28 (ESI+)	0.6832	0.7480	0.7069	0.7977	0.7689	0.7484	0.8027	0.8225	0.7947	0.8076
	MTBLS28 (ESI-)	0.6329	0.7138	0.7162	0.7097	0.7738	0.7532	0.7661	0.7645	0.7576	0.7967
	MTBLS17 (ESI+)	0.5385	0.4103	0.6282	0.4744	0.6154	0.6154	0.6026	0.6923	0.6795	0.7564
	MTBLS17 (ESI-)	0.6113	0.7955	0.8725	0.8563	0.8381	0.8968	0.9372	0.9575	0.9352	0.9170
Range Scaling	MTBLS28 (ESI+)	0.6775	0.7460	0.7778	0.7355	0.7617	0.7182	0.7633	0.7981	0.7834	0.7862
	MTBLS28 (ESI-)	0.6647	0.6445	0.6242	0.6707	0.7089	0.6916	0.7045	0.6896	0.7206	0.7238
	MTBLS17 (ESI+)	0.5513	0.4744	0.5641	0.6538	0.7308	0.6410	0.7436	0.6410	0.5641	0.7308
	MTBLS17 (ESI-)	0.6822	0.7530	0.8704	0.6781	0.7976	0.9231	0.8158	0.7227	0.8785	0.8947
Vast Scaling	MTBLS28 (ESI+)	0.6852	0.7613	0.7943	0.7335	0.7709	0.7440	0.7689	0.7967	0.7798	0.7729
	MTBLS28 (ESI-)	0.6574	0.6381	0.6433	0.7158	0.7399	0.7122	0.7208	0.7118	0.7436	0.7323
	MTBLS17 (ESI+)	0.5513	0.4615	0.4615	0.5897	0.6923	0.5641	0.7179	0.6667	0.6410	0.7564
	MTBLS17 (ESI-)	0.6862	0.7348	0.8704	0.7368	0.7713	0.9028	0.8198	0.7389	0.8846	0.8947
VSN	MTBLS28 (ESI+)	0.7470	0.7653	0.7528	0.8092	0.7721	0.8200	0.8325	0.7903	0.8543	0.8535
	MTBLS28 (ESI-)	0.6940	0.7709	0.7717	0.7774	0.7792	0.7838	0.7955	0.8253	0.8092	0.8104
	MTBLS17 (ESI+)	0.5256	0.5256	0.6410	0.6154	0.6923	0.7949	0.7308	0.8077	0.8077	0.7308
	MTBLS17 (ESI-)	0.6640	0.8279	0.8988	0.8745	0.8684	0.9089	0.9190	0.9291	0.9919	0.9413

Supplementary NOTES

Note S1. Description of 16 normalization methods applied in this study

Auto Scaling (unit variance scaling, UV)

The methods aimed at adjusting the variance of different metabolites include variable scaling and variance stabilization approaches. The simplest of these approaches uses the standard deviation of the data as a scaling factor. This method is called Auto Scaling or unit variance (UV) scaling. The equation used in Auto Scaling was defined as:

$$\tilde{x}_{ij} = \frac{x_{ij} - \bar{x}_i}{s_i} \tag{1}$$

where s_i represents the standard deviation of bucket$_i$. The method will result in a fluctuation of the data around zero, thereby adjusting for offsets between high and low intensity features. But measurement errors will also be inflated, and between sample variations due to dilution effects, which in case of urine spectra are caused, for example, by variations in fluid intake will not be corrected.

Contrast Normalization (Contrast)

Contrast Normalization uses MA-plots and makes the same assumptions as Cyclic Loess. The data matrix uses the alternative matrix:

$$\mathrm{Z} = \log(\mathrm{X}) \cdot \mathrm{T} \tag{2}$$

$$z_{ij} = \sum_{k=1}^{J} log\,(x_{ik}) m_{kj} = \left[\vec{x}_{sum,}\vec{x}_{cont_1}, \dots \vec{x}_{cont_{J-1}}\right] \tag{3}$$

where $\mathrm{T} = \left(\mathrm{t_{ij}}\right)$ is the orthonormal transformation matrix.

Then evaluating contrasts by computing the multi-loess fits $\left[\hat{\vec{x}}_{cont_1}, \dots \hat{\vec{x}}_{cont_{J-1}}\right]$, using the Euclidean distance $\epsilon = \sqrt{\sum_{j=1}^{J-1} \left(\hat{\vec{x}}_{cont_j} - \vec{x}_{cont_j}\right)^2}$.

Loess $\left[\tilde{\vec{x}}_{sum}, \tilde{\vec{x}}_{cont_1}, \dots \tilde{\vec{x}}_{cont_{J-1}}\right]$ map smoothly from the contrasts to zero:

$$\left[\vec{x}_{sum}, \hat{\vec{x}}_{cont_1}, \dots \hat{\vec{x}}_{cont_{J-1}}\right] \cdot T \mapsto [\vec{x}_{sum}, 0, \dots 0] \cdot \mathrm{T} \tag{4}$$

This expands the idea of MA-plots to several dimensions and converts the data into a set of rows representing orthonormal contrasts. But the use of a log function impedes the handling of negative values and zeros.

Cubic Splines

Another non-linear baseline method makes use of Cubic Splines. As in quantile normalization the aim is to obtain a similar distribution of feature intensities across spectra. The equation used in Cubic Splines was defined as:

$$x_{target_i} = \frac{1}{J}\sum_{j=1}^{J} x_{ij} \text{ (target array)} \tag{5}$$

Evenly spaced set of N quantiles of the target array and sample j: $j: (x_{target_n}, x_{jn})_{n=1 \ldots N}$

The use of a cubic spline generator for each iteration k = 1…K: $c_{jk} = f(x_{target_n}, x_{jn})$ leads to the interpolated spline $s_j = \frac{1}{K}\sum_{k=1}^{K} c_{jk}$. In the n-th interval, the spline of sample j is defined by:

$$s_{jn}(x) = a_{jn_1} + a_{jn_2}\left(x - x_{target_n}\right) + a_{jn_3}\left(x - x_{target_n}\right)^2 + a_{jn_4}\left(x - x_{target_n}\right)^3 \tag{6}$$

where $\vec{a}_{jn} = [a_{jn_1}, a_{jn_2}, a_{jn_3}, a_{jn_4}]$. The normalized intensities are: $\tilde{x}_{ij} = s_j(x_{ij})$. Moreover, a set of evenly distributed quantiles is taken from both the target spectrum and the sample spectrum and used to fit a smooth cubic spline.

Cyclic Locally Weighted Regression (Cyclic Loess)

Cyclic Loess is based on MA-plots, which constitute logged Bland-Altman plots. The equation used in Cyclic Loess was defined as:

$$M_{ij_1j_2} = log_2\left(x_{ij_1}\right) - log_2\left(x_{ij_2}\right) = log_2(\frac{x_{ij_1}}{x_{ij_2}}) \tag{7}$$

$$A_{ij_1j_2} = \frac{1}{2}\left(log_2\left(x_{ij_1}\right) + log_2\left(x_{ij_2}\right)\right) = \frac{1}{2} log_2\left(x_{ij_1}x_{ij_2}\right) \tag{8}$$

Normalizing a pair of samples j_1 and j_2: $\tilde{x}_{ij_1} = 2^{A_k + \frac{\widetilde{M}_k}{2}}$, $\tilde{x}_{ij_2} = 2^{A_k + \frac{\widetilde{M}_k}{2}}$, where $\widetilde{M}_K = M_k - M_{k,fit}$. Repeat it with all $\frac{J(J-1)}{2}$ pairs of samples. If more than two spectra need to be normalized, the method is iterated in pairs for all possible combinations. Here, all data points are used.

Level Scaling

Level Scaling focuses on relative response. The equation used in Vast Scaling was defined as:

$$\tilde{x}_{ij} = \frac{x_{ij} - \bar{x}_i}{\bar{x}_i} \tag{9}$$

It suits for identification of e.g. biomarkers. But it also has a weakness that inflation of the measurement errors.

Linear Baseline Scaling (Linear Baseline)

Linear Baseline Scaling uses a scaling factor to map linearly from each spectrum to the baseline. It assumes a constant linear relationship between each feature of a given spectrum and the baseline. The equation used in the Linear Baseline was defined as:

$$\tilde{x}_{ij} = \beta_j x_{ij} \tag{10}$$

where $\beta_j = \frac{\bar{x}_{baseline}}{\bar{x}_j}$. However, the assumption of a linear correlation between spectra may constitute an oversimplification.

Log Transformation

Log Transformation is a method for data transformation, and it can be used to make highly skewed distributions less skewed. This can be valuable both for making patterns in the data more interpretable and for helping to meet the assumptions of inferential statistics. The equation used in Log Transformation was defined as:

$$\tilde{x}_{ij} = \log_{10} x_{ij} - \frac{\sum_1^j \log_{10} x_{ij}}{j} \tag{11}$$

Notes: The original data were represented by $X = x_{ij}$, and the normalized data by $\tilde{X} = \tilde{x}_{ij}$. Additionally, the mean is estimated as:

$$\bar{x}_i = \frac{1}{J}\sum_{j=1}^{J} x_{ij} \tag{12}$$

The standard deviation is estimated as:

$$S_i = \sqrt{\frac{\sum_{j=1}^{J}(x_{ij}-\bar{x}_i)^2}{J-1}} \tag{13}$$

$\tilde{x}$ and $\hat{x}$ represent the data after different pretreatment steps.

MS Total Useful Signal (MSTUS)

MSTUS uses the total intensity of metabolites that are common to all samples assuming that there are similar numbers of metabolites with increased and decreased signals. Its normalization factor was calculated by summing the peak areas for markers common to all samples. As a result, each sample had its own normalization factor based only upon the total intensities of peaks which are also common to all other samples, thus eliminating any bias as a result of xenobiotic intake or random background noise. All peaks

areas within a chromatogram were subsequently divided by the sample MSTUS normalization factor. The equation used in MSTUS was defined as:

$$x_{ij} = \frac{x_{ij}}{\sum_1^J x_{ij}} \quad (14)$$

The x_{ij} represents the peak intensity of any peak in given chromatogram, and the $\sum_1^J x_{ij}$ represents the sum peak intensity of all common peaks in the same chromatogram.

Non-Linear Baseline Normalization (Li-Wong)

A more complex approach is to fit a Non-Linear Baseline Normalization relationship between the spectra that are to be normalized and the baseline as implemented by Li and Wong. It is assumed that features corresponding to unregulated metabolites have similar intensity ranks in two spectra, allowing a reliable determination of a normalization curve. The equation used in Li-Wong is defined as:

$$x_{i,baseline} = x_{i,j_{median}} \quad (15)$$

where $\bar{x}_{j_{median}} = median(x_i, \ldots x_J)$. Based on the proportion rank difference (PRD), finding a set of rank-invariant features k between baseline and each sample: $(x_{k,baseline,}\ x_{kj})$. Then calculating piecewise linear running median line based on k $m_{kj}(x) = m(x_{k,baseline}, x_{kj})$ used as normalization curve $\tilde{x}_{ij} = m_{kj}(x_{ij})$. Ideally, the data should align along the diagonal y = x. As the non-normalized data generally deviates from that, the normalization curve is then fitted to map the data to the diagonal.

Pareto Scaling

Using the square root of the standard deviation is an alternative used by Pareto Scaling. It is similar to Auto Scaling, but its normalizing effect is less intense, such that the normalized data stays closer to its original values. The equation used in Pareto Scaling was defined as:

$$\tilde{x}_{ij} = \frac{x_{ij} - \bar{x}_i}{\sqrt{s_i}} \quad (16)$$

It is less likely to blow up noisy background and reduces the importance of large fold changes compared to small ones. But very large fold changes may still show a dominating effect.

Power Scaling

Power Scaling is one of the remedial actions that may help to make data normal. The equation used in Power Scaling was defined as:

$$\tilde{x}_{ij} = \sqrt{x_{ij}} - \frac{\sum_1^j \sqrt{x_{ij}}}{j} \quad (17)$$

Probabilistic Quotient Normalization (PQN)

PQN assumes that biologically interesting changes in concentration influence only parts of the NMR spectrum, while dilution effects will affect all metabolite signals. The reference spectrum is defined as:

$$x_{ref,i} = \frac{1}{J}\sum_{j=1}^{J} x_{ij} \tag{18}$$

For each spectrum, the equation used for calculating the quotients of all features each spectrum to the reference spectrum is defined as:

$$q_{ij} = \frac{x_{ij}}{x_{ref,i}} \tag{19}$$

The median of the quotients represents the scaling factor:

$$q_{med,j} = med(q_i)_j \tag{20}$$

The normalized intensities are defined as:

$$\tilde{x} = \frac{x_{ij}}{q_{med.j}} \tag{21}$$

All variables of the test spectrum are divided by the median quotient. But in case of urine spectra, dilution effects are caused, for example, by variations in fluid intake.

Quantile Normalization (Quantile)

The goal of Quantile Normalization is to achieve the same distribution of feature intensities across all spectra. Similarity of distributions can be visualized in a quantile-quantile plot. The equation used in the Quantile Normalization was defined as:

$$\tilde{x}_{ij} = \frac{1}{J}\sum_{l=1}^{J} x_{k_l,l} \tag{22}$$

where k_l such that rank$\left(x_{k_l,l}\right) = rank\left(x_{ij}\right), \forall\ l = 1 \ldots J$. The idea is to bring simply all spectra to an identical distribution of intensities across features (bins). Since different features may display the highest intensity in different samples, this constant average value may be assigned to different features across samples.

Range Scaling

The equation used in Range Scaling was defined as:

$$\tilde{x}_{ij} = \frac{x_{ij} - \bar{x}_i}{x_{i_{max}} - x_{i_{min}}} \tag{23}$$

In this method, all metabolites become equally important. Scaling is related to biology. But it also has a weakness that inflation of the measurement errors and sensitive to outliers.

Variance Stabilization Normalization (VSN)

VSN transformations are a set of non-linear methods that aim to keep the variance constant over the entire data range. The equation used in VSN was defined as:

$$\tilde{x}_{ij} = arsinh(a_j + b_j x_{ij}) \tag{24}$$

$$arsinh(t) = \log\left(t + \sqrt{t^2 + 1}\right) \tag{25}$$

where the 2 parameters a_j and b_j are determined using a robust maximun likelihood estimator such that the variance is constant. This transformation approaches the logarithm for large values, therefore removing heteroscedasticity. As values approach the lower limit of detection, variance does not decrease any more, but rather stays constant, thus, the coefficient of variation increases.

Vast Scaling

Vast Scaling focuses on the metabolites that show small fluctuations. The equation used in Vast Scaling was defined as:

$$\tilde{x}_{ij} = \frac{(x_{ij} - \bar{x}_i)}{s_i} \cdot \frac{\bar{x}_i}{s_i} \tag{26}$$

This method aims for robustness, and can use prior group knowledge. But it isn’t suited for large inducted variation without group structure.

Note S2. All source codes of programs designed in this study

Source code of program 1: preprocessing datasets and sampling from local folder

```
#-- In the Following, the R code for the normalizations used in the paper is given.

#-- The non-normalized data matrix of feature intensities is stored in 'data'.

#-- Each row represents a feature, each column a sample.

### Step 1: Preprocessing MTBLS28 raw data using xcms package in R.

setwd("/Users/idrb/test")

library(xcms)

cdffile <- list.files("./POS", recursive = TRUE, full.names = TRUE)

xr <- xcmsSet(cdffile)

xsg <- group(xr, bw = 10, sleep = 1e-04)

xsr <- retcor(xsg, method = "obiwarp")

xsg <- group(xsr, bw = 10)

xsg <- fillPeaks(xsg)

data <- groupval(xsg, "maxint", "into")

write.csv(data, file = "mtblst28.csv")

### Step 2: Generating of Benchmark dataset and sub-datasets.

### 2.1 - All 1005 samples were divided into training group and independent testing group by random selection,
i. e., holdout validation.

### In the object of matrix or data frame, the first column and the second column were names of sample and
labels of sample, respectively.

library(openxlsx)

data <- read.csv("mtblst28_label.csv", header = TRUE)

control_sample <- sample(data[data[, 2] == 0, 1], 36)

ord_con <- match(control_sample, data[, 1])

controldata <- data[ord_con, ]
```

```
case_sample <- sample(data[data[, 2] == 1, 1], 69)

ord_case <- match(case_sample, data[, 1])

casedata <- data[ord_case, ]

allvali <- c(ord_con, ord_case)

alltrain_data <- data[-allvali, ]

allvali_data <- data[allvali, ]

## Independent testing group include 105 samples.

write.xlsx(allvali_data, file = "IndependentTest(105samples).xlsx")

## Training group include 900 samples.

write.xlsx(alltrain_data, file = "./train_data/percent100.xlsx")

### 2.2 - Generating the sub-datasets (10 datasets) from the training group using K-means clustering.

dat_con <- alltrain_data[alltrain_data[, 2] == 0, ]

dat_case <- alltrain_data[alltrain_data[, 2] == 1, ]

dat_con <- dat_con[, -(1:2)]

dat_case <- dat_case[, -(1:2)]

con_grad <- seq(0.1, 0.9, 0.1) * 500

case_grad <- seq(0.1, 0.9, 0.1) * 400

## (1) Generating 10%, 20%, 30%, 40%, 50%, 60%, 70%, 80%, 90% of the control in training group,
respectively.

sub_dat_con <- list()

for (i in 1:length(con_grad)) {

        fit_kml <- kmeans(dat_con, center = con_grad[i], iter.max = 10, nstart = 1)

        k <- as.data.frame(fit_kml$cluster)

        data <- cbind(k, rownames(k))

        colnames(data)[2] <- "sample.order"

        ff <- NULL

        for (j in 1:con_grad[i]) {
```

```
specific_cluster <- data[data[, 1] == j, ]
specific_cluster_sample <- specific_cluster[, 2]
hh <- NULL
for (h in 1:length(specific_cluster_sample)) {
from_g_dis <- dist(rbind(dat_con[which(rownames(dat_con) ==
specific_cluster_sample[h]), ],
fit_kml$centers[specific_cluster[1, 1], ]),
method = "euclidean")
from_g_dis <- as.numeric(from_g_dis)
hh[h] <- list(from_g_dis)
}
res <- specific_cluster[which.min(hh), ]
result <- as.vector(res[, 2])
ff[j] <- list(result)
}
ll <- match(unlist(ff), rownames(dat_con))
resultdata <- dat_con[ll, ]
sub_dat_con[[i]] <- list(resultdata)
}
save(sub_dat_con, file = "sub_dat_con.Rdata")
## (2) Generating 10%, 20%, 30%, 40%, 50%, 60%, 70%, 80%, 90% of the case in training group, respectively.
sub_dat_case <- list()
for (i in 1:length(case_grad)) {
fit_kml <- kmeans(dat_case, center = case_grad[i], iter.max = 10, nstart = 1)
k <- as.data.frame(fit_kml$cluster)
data <- cbind(k, rownames(k))
colnames(data)[2] <- "sample.order"
```

```
        ff <- NULL
        for (j in 1:case_grad[i]) {
                specific_cluster <- data[data[, 1] == j, ]
                specific_cluster_sample <- specific_cluster[, 2]
                hh <- NULL
                for (h in 1:length(specific_cluster_sample)) {
                        from_g_dis <- dist(rbind(dat_case[which(rownames(dat_case) ==
specific_cluster_sample[h]), ], fit_kml$centers[specific_cluster[1, 1], ]),
                                           method = "euclidean")
                        from_g_dis <- as.numeric(from_g_dis)
                        hh[h] <- list(from_g_dis)
                }
                res <- specific_cluster[which.min(hh), ]
                result <- as.vector(res[, 2])
                ff[j] <- list(result)
        }
        ll <- match(unlist(ff), rownames(dat_case))
        resultdata <- dat_case[ll, ]
        sub_dat_case[[i]] <- list(resultdata)
}
save(sub_dat_case, file = "sub_dat_case.Rdata")
setwd("./train_data")
for (i in 1:9) {
        sub_data <- rbind(as.data.frame(sub_dat_con[i][[1]]),
                          as.data.frame(sub_dat_case[i][[1]]))
        label <- c(rep(0, con_grad[i]), rep(1, case_grad[i]))
        percentage <- cbind(sub_data, label = label)
```

```
        file_name <-    paste0("percent", formatC(i, format = "fg", digits = 1,
                                                    flag = "0"), "0.xlsx")
        write.xlsx(percentage, file = file_name)
}
setwd("..")
```

Source code of program 2: all normalization methods applied in this study

```
### ---------------------------- Method - 01 --------------------------------###
### Auto Scaling (unit variance scaling).
AUTO <- function(data) {
        centered.data <- data - apply(data, 1, mean)
        scaling.auto <- apply(data, 1, sd)
        auto.data <- centered.data / scaling.auto
        return(auto.data)
}
### ---------------------------- Method - 02 --------------------------------###
### Contrast Normalization (Contrast).
# Load package unless it is already loaded
library(affy)
CONTRAST <- function(data) {
        #---First adaption: Make the data matrix non-negative
        threshold = 1e-11
        data[data <= 0] <- threshold
        #---Apply normalization
        maffy.data <- maffy.normalize(data, subset = 1:nrow(data), span = 0.75,
                                      verbose = TRUE,
                                      family = "gaussian", log.it = FALSE)
```

```
        #---Second adaption: Subtract 10% Quantile from each sample
        subtract <- function(x) {
                t(t(x) - apply(x, 2, quantile, 0.1))
        }
        contrast.data <- subtract(maffy.data)
        return(contrast.data)
}
### ---------------------------- Method - 03 --------------------------------###
###    Cubic Spline Normalization (Cubic Spline).
CUBIC <- function(data) {
        # load package unless it is already loaded
        library(affy)
        spline.data <- normalize.qspline(data, samples = 0.02,
                                         target = apply(data, 1, mean))
        return(spline.data)
}
### ---------------------------- Method - 04 --------------------------------###
### Cyclic Locally Weighted Regression (Cyclic Loess Normalization).
# load package unless it is already loaded
library(affy)
LOESS <- function(data) {
        loess.data <- normalize.loess(data, subset = 1:nrow(data),
                                      epsilon = 10^-2, maxit = 2,
                                      log.it = FALSE, verbose = TRUE,
                                      span = 0.75, family.loess = "gaussian")
        return(loess.data)
}
```

```
### ---------------------------- Method - 05 --------------------------------###
### Level Scaling.
LEVEL <- function(data) {
        level.data <- scalingMethods(data, methods = "level")
        return(level.data)
}
### ---------------------------- Method - 06 --------------------------------###
### Linear Baseline Normalization (Linear Baseline).
LINEAR <- function(data) {
        linear.baseline <- apply(data, 1, median)
        baseline.mean <- mean(linear.baseline)
        sample.means <- apply(data, 2, mean)
        linear.scaling <- baseline.mean/sample.means
        linear.baseline.data <- t(t(data) * linear.scaling)
        return(linear.baseline.data)
}
### ---------------------------- Method - 07 --------------------------------###
### Log Transformation.
LOGTRAN <- function(data) {
        tmp_data <- apply(data, 1, function(x) log10(x) - mean(log10(x)))
        logtran.data <- data.frame(t(tmp_data), check.names = FALSE)
        return(logtran.data)
}
### ---------------------------- Method - 08 --------------------------------###
### non-Linear Baseline Normalization (Li-Wong Normalization).
# load package unless it is already loaded
library(affy)
```

```
LIWONG <- function(data) {
	#---First step: Find baseline sample
	average.intensity <- apply(data, 2, mean)
	# R has an add way of rounding.
	median.number <- round(ncol(data) / 2 + 0.1)
	# the additional 0.1 ensures that it rounds properly
	ordering <- order(average.intensity)
	median.sample.number <- ordering[median.number]
	median.sample <- data[, median.sample.number]
	#---Apply normalization
	liwong.data <- vector()
	for (i in 1:ncol(data)) {
		liwong.model <- normalize.invariantset(data = data[, i],
		                                       ref = median.sample,
		                                       prd.td = c(0.003, 0.007))
	# the threshold of the rank-invariant set might need to be adjusted from case to case.
		liwong.sample <- predict(liwong.model$n.curve$fit, data[, i])
		liwong.data <- cbind(liwong.data, liwong.sample$y)
	}
	return(liwong.data)
}
### ---------------------------- Method - 09 --------------------------------###
###	Pareto Scaling.
PARETO <- function(data) {
	centered.data <- data - apply(data, 1, mean)
	scaling.pareto <- sqrt(apply(data, 1, sd))
	pareto.data <- centered.data / scaling.pareto
```

```r
        return(pareto.data)
}
### ---------------------------- Method - 10 --------------------------------###
### Power Scaling.
POWER <- function(data) {
        power.data <- scalingMethods(data, methods = "power")
        return(power.data)
}
### ---------------------------- Method - 11 --------------------------------###
### Probabilistic Quotient Normalization (PQN).
PQN <- function(data) {
        reference <- apply(data, 1, median)
        quotient <- data / reference
        quotient.median <- apply(quotient, 2, median)
        pqn.data <- t(t(data) / quotient.median)
        return(pqn.data)
}
### ---------------------------- Method - 12 --------------------------------###
### Quantile Normalization (Quantile).
# load package unless it is already loaded
library(affy)
QUANTILE <- function(data) {
        normalize.quantile <- get("normalize.quantiles", en = asNamespace("affy"))
        quantile.data <- normalize.quantile(data)
        return(quantile.data)
}
### ---------------------------- Method - 13 --------------------------------###
```

```
### Range Scaling.
RANGE <- function(data) {
        if (!require("DiffCorr") == TRUE)
                biocLite("DiffCorr")
        range.data <- scalingMethods(data, methods = "range")
        return(range.data)
}
### ---------------------------- Method - 14 --------------------------------###
### Variance Stabilization Normalization (VSN).
# load package unless it is already loaded
VSN <- function(data) {
        library(vsn)
        vsn.model <- vsn2(data)
        vsn.data <- predict(vsn.model, data)
        return(vsn.data)
}
### ---------------------------- Method - 15 --------------------------------###
### Vast Scaling.
VAST <- function(data) {
        vast.data <- scalingMethods(data, methods = "vast")
        return(vast.data)
}
```

Source code of program 3: marker selection methods applied in this study

```
### Defining the approach for marker selection.
### For this method, PLS-DA and Student t-test were jointly used.
#-- input1: mat, a matrix with sampple in column and variable in row.
```

```
#-- input2: label with vector attributes.
library(ropls)
markerSelect <- function(data) {
        OPLSDA_test <- function(mat, label) {
                X <- t(mat)
                Y <- as.factor(label)
                oplsda <- opls(X, Y, permI = 100)
                res <- oplsda$vipVn
                cpds <- data.frame(CompoundName = names(res), VIP = res)
                return(cpds)
        }
        opls_res <- OPLSDA_test(data, train_label)
        rowTTest <- function(x) {
                s2e <- 1:(length(train_label) * 5 / 9)
                t_res <- t.test(x[s2e], x[-s2e], paired = FALSE)
                return(t_res$p.value)
        }
        ttest_res <- apply(data, 1, rowTTest)
        marker_matrix <- data[opls_res$VIP > 1 & ttest_res < 0.05, ]
        return(marker_matrix)
}
```

Source code of program 4: marker Selection, construction of SVM model and ROC.

```
indep_set <- openxlsx::read.xlsx(xlsxFile = "IndependentTest(105samples).xlsx",
                                 sheet = 1, startRow = 1, colNames = TRUE,
                                 rowNames = TRUE)
indep_label <- indep_set$Label
```

```
indep_set <- indep_set[, -1]
setwd("./plot")
library(ROCR)
PQN_r <- list()
for (i in 1:10) {
	datao <- openxlsx::read.xlsx(xlsxFile = file[i], sheet = 1, startRow = 1,
					colNames = TRUE, rowNames = TRUE)
	train_data <- t(datao[, -1])
	train_label <- datao[, 1]
	# save(train_data, file = "train_data.RData")
	# load("train_data.RData")
	PQN_data <- PQN(train_data)
	PQN_indep <- PQN(t(indep_set))
	x4svm <- markerSelect(PQN_data)
	X <- t(x4svm)
	train_label <- as.factor(train_label)
	obj <- tune(svm, X, train_label, ranges = list(gamma = 10^(-5:5),
							cost = 2^(0:2)),
		tunecontrol = tune.control(sampling = "cross"))
	# Best gamma.
	best_gm <- obj$best.parameters$gamma
	best_gm
	# Best C-value.
	best_ct <- obj$best.parameters$cost
	best_ct
	PQN_test <- t(PQN_indep[rownames(x4svm), ])
	model <- svm(X, train_label, probability = TRUE, scale = TRUE,
```

```
                                kernel = "radial", gamma = best_gm, cost = best_ct, cross = 0)
        pre_label <- predict(model, PQN_test, probability = TRUE)
        stat_res <- table(pre_label, indep_label)
        accurary <- (stat_res[1, 1] + stat_res[2, 2])/length(pre_label)
        sensitivity <- stat_res[2, 2]/(stat_res[1, 2] + stat_res[2, 2])
        specificity <- stat_res[1, 1]/(stat_res[1, 1] + stat_res[2, 1])
        pre_prob <- attr(pre_label, "probabilities")
        par(mfrow = c(1, 1))
        pred <- prediction(pre_prob[, 2], indep_label)
        perf <- performance(pred, "tpr", "fpr")
        auc.tmp <- performance(pred, "auc")
        auc.value <- auc.tmp@y.values
        AUC <- round(auc.value[[1]], digits = 4)
        plot(perf, colorize = TRUE, main = paste("AUC=", round(auc.value[[1]],
                                                          digits = 4) * 100,
                                                 "%", sep = ""))
        grid(5, 5, lwd = 1)
        lines(c(0, 1), c(0, 1), lty = 2, lwd = 2, col = "red")
        tj <- c(dim(X)[2], best_gm, best_ct, accurary, sensitivity, specificity, AUC)
        PQN_r[i] <- list(tj)
}
save(PQN_r, file = "PQN_r.RData")
save(perf, file = "PQN_perf.RData")
setwd("..")
```

Source code of program 5: assessment by ROC and accuracy.

```
### Step 1: Comparison among the classification performances of 15 normalization methods
```

```r
### Measured by the receiver operating characteristic (ROC) curves.

library(ROCR)

setwd("./plot")

load("AUTO_perf.RData")

x <- unlist(perf@x.values)

y <- unlist(perf@y.values)

lo <- loess(y ~ x)

plot(x, y, col = "white", xlab = "1-Specificity", ylab = "Sensitivity")

lines(x, predict(lo), lwd = 3, lty = 1, col = "black")

load("CUBIC_perf.RData")

x <- unlist(perf@x.values)

y <- unlist(perf@y.values)

lo <- loess(y ~ x)

lines(x, predict(lo), lwd = 3, col = "darkorchid")

load("LEVEL_perf.RData")

x <- unlist(perf@x.values)

y <- unlist(perf@y.values)

lo <- loess(y ~ x)

lines(x, predict(lo), lwd = 3, col = "red")

load("PARETO_perf.RData")

x <- unlist(perf@x.values)

y <- unlist(perf@y.values)

lo <- loess(y ~ x)

lines(x, predict(lo), lwd = 3, col = "steelblue1")

load("POWERperf.Rdata")

x <- unlist(perf@x.values)

y <- unlist(perf@y.values)
```

```
lo <- loess(y ~ x)
lines(x, predict(lo), lwd = 3, col = "orange")
load("RANGE_perf.RData")
x <- unlist(perf@x.values)
y <- unlist(perf@y.values)
lo <- loess(y ~ x)
lines(x, predict(lo), lwd = 3, col = "springgreen4")
load("VAST_perf.RData")
x <- unlist(perf@x.values)
y <- unlist(perf@y.values)
lo <- loess(y ~ x)
lines(x, predict(lo), lwd = 3, col = "yellow3")
load("VSNperf.Rdata")
x <- unlist(perf@x.values)
y <- unlist(perf@y.values)
lo <- loess(y ~ x)
lines(x, predict(lo), lwd = 3, col = "mediumblue")
load("LINEARpref.Rdata")
x <- unlist(perf@x.values)
y <- unlist(perf@y.values)
lo <- loess(y ~ x)
lines(x, predict(lo), lwd = 3, col = "red", lty = 2)
load("CONTRASTpref.Rdata")
x <- unlist(perf@x.values)
y <- unlist(perf@y.values)
lo <- loess(y ~ x)
lines(x, predict(lo), lwd = 3, col = "steelblue1", lty = 2)
```

```r
load("LIWONGperf.Rdata")
x <- unlist(perf@x.values)
y <- unlist(perf@y.values)
lo <- loess(y ~ x)
lines(x, predict(lo), lwd = 3, col = "springgreen4", lty = 2)
load("loessperf.Rdata")
x <- unlist(perf@x.values)
y <- unlist(perf@y.values)
lo <- loess(y ~ x)
lines(x, predict(lo), lwd = 3, col = "mediumblue", lty = 2)
load("logtranperf.Rdata")
x <- unlist(perf@x.values)
y <- unlist(perf@y.values)
lo <- loess(y ~ x)
lines(x, predict(lo), lwd = 3, col = "yellow3", lty = 2)
load("PQNperf.Rdata")
x <- unlist(perf@x.values)
y <- unlist(perf@y.values)
lo <- loess(y ~ x)
lines(x, predict(lo), lwd = 3, col = "darkorchid", lty = 2)
load("QUANTILEperf.Rdata")
x <- unlist(perf@x.values)
y <- unlist(perf@y.values)
lo <- loess(y ~ x)
lines(x, predict(lo), lwd = 3, col = "orange", lty = 2)
lines(c(0, 1), c(0, 1), lty = 1, lwd = 2, col = "grey83")
legend = c("AUTO(AUC:0.7806)", "Cubic Spline(AUC:0.7802)", "LEVEL(AUC:0.8015)",
```

```
                "Pareto(AUC:0.7697)", "Power(AUC:0.7822)", "Range(AUC:0.7307)",
                "VAST(AUC:0.7802)", "VSN(AUC:0.8269)", "Linear Baseline(AUC:0.7576)",
                "Contrast(AUC:0.5076)", "Li-Wong(AUC:0.7568)", "Cyclic Loess(AUC:0.7854)",
                "Log transf(AUC:0.8378)", "PQN(AUC:0.7202)", "Quantile(AUC:0.779)")
legend("bottomright", legend = legend,
       col = c("black", "darkorchid", "red", "steelblue1", "orange",
               "springgreen4", "yellow3", "mediumblue", "red", "steelblue1",
               "springgreen4", "mediumblue", "yellow3", "darkorchid", "orange"),
       lwd = 2, lty = c(rep(1, 8), rep(2, 7)),
       cex = 0.7, seg.len = 2, text.width = 0.28)
setwd("..")
### Step 2: The dependence of classification performance of each normalization method on the sample size
### Reading AUC values matrix of biomarkers identified across 15 different methods in 10 subsamples.
library(ROCR)
AUCs <- read.csv("AUC_matrix", header = FALSE)
rn <- AUCs[, 1]
AUCs <- AUCs[, -1]
rownames(AUCs) <- rn
cn <- seq(90, 900, by = 90)
colnames(AUCs) <- cn
par(mfrow = c(1, 1), bg = "white")
x <- 1:10
# Auto Scaling
y <- unlist(AUCs[1, ])
lo <- loess(y ~ x)
plot(x, y, col = "white", xlab = "Sample size",
     ylab = "AUC values", ylim = c(0.46, 0.91),
```

```r
        xaxt = "n")
lines(x, predict(lo), lwd = 3, lty = 1, col = "black")
# Contrast
y <- unlist(AUCs[2, ])
lo <- loess(y ~ x)
lines(x, predict(lo), lwd = 3, col = "steelblue1", lty = 2)
# Cubic Splines
y <- unlist(AUCs[3, ])
lo <- loess(y ~ x)
lines(x, predict(lo), lwd = 3, col = "darkorchid")
# Cyclic Loess
y <- unlist(AUCs[4, ])
lo <- loess(y ~ x)
lines(x, predict(lo), lwd = 3, col = "mediumblue", lty = 2)
# Level Scaling
y <- unlist(AUCs[5, ])
lo <- loess(y ~ x)
lines(x, predict(lo), lwd = 3, col = "red")
# Li-Wong
y <- unlist(AUCs[6, ])
lo <- loess(y ~ x)
lines(x, predict(lo), lwd = 3, col = "springgreen4", lty = 2)
# Linear Baseline
y <- unlist(AUCs[7, ])
lo <- loess(y ~ x)
lines(x, predict(lo), lwd = 3, col = "red", lty = 2)
# Log Transformation
```

```r
y <- unlist(AUCs[8, ])
lo <- loess(y ~ x)
lines(x, predict(lo), lwd = 3, col = "yellow3", lty = 2)
# Pareto Scaling
y <- unlist(AUCs[9, ])
lo <- loess(y ~ x)
lines(x, predict(lo), lwd = 3, col = "steelblue1")
# Power Scaling
y <- unlist(AUCs[10, ])
lo <- loess(y ~ x)
lines(x, predict(lo), lwd = 3, col = "orange")
# PQN
y <- unlist(AUCs[11, ])
lo <- loess(y ~ x)
lines(x, predict(lo), lwd = 3, col = "darkorchid", lty = 2)
# Quantile
y <- unlist(AUCs[12, ])
lo <- loess(y ~ x)
lines(x, predict(lo), lwd = 3, col = "orange", lty = 2)
# Range Scaling
y <- unlist(AUCs[13, ])
lo <- loess(y ~ x)
lines(x, predict(lo), lwd = 3, col = "springgreen4")
# Vast Scaling
y <- unlist(AUCs[14, ])
lo <- loess(y ~ x)
lines(x, predict(lo), lwd = 3, col = "yellow3")
```

```r
# VSN
y <- unlist(AUCs[15, ])
lo <- loess(y ~ x)
lines(x, predict(lo), lwd = 3, col = "mediumblue")
axis(1, at = 1:10, c(90, 180, 270, 360, 450, 540, 630, 720, 810, 900))
legend = c("AUTO", "Cubic Spline", "LEVEL", "Pareto", "Power", "Range",
           "VAST", "VSN", "Linear Baseline", "Contrast", "Li-Wong",
           "Cyclic Loess", "Log transf", "PQN", "Quantile")
legend("bottomright", legend = legend,
       col = c("black", "darkorchid", "red", "steelblue1", "orange",
               "springgreen4", "yellow3", "mediumblue", "red", "steelblue1",
               "springgreen4", "mediumblue", "yellow3", "darkorchid", "orange"),
       lwd = 2, lty = c(rep(1, 8), rep(2, 7)),
       cex = 0.55, horiz = FALSE)
### Step 3: The identified 4 groups of 15 normalization methods identification in 10 sub-samples.
### Divided into four pictures.
op <- par(mfrow=c(2, 2), bg = "white")
x <- 1:10
### (A) Superior performance group
### VSN.
y <- unlist(AUCs[15, ])
lo <- loess(y ~ x)
plot(x, y, col = "white", xlab = "Sample size", ylab = "AUC values",
     ylim = c(0.46, 0.91), xaxt="n",
     main = "A. Superior performance group")
lines(x, predict(lo), lwd = 3, lty = 1, col = "mediumblue")
axis(1, at = 1:10, paste0(seq(90, 900, by = 90)))
```

```
### Log Transformation.
y <- unlist(AUCs[8, ])
lo <- loess(y ~ x)
lines(x, predict(lo), lwd = 3, col = "yellow3", lty = 2)
### (B) Consistently good performance group
### Auto Scaling.
y <- unlist(AUCs[1, ])
lo <- loess(y ~ x)
plot(x, y, col = "white", xlab = "Sample size", ylab = "AUC values",
     ylim = c(0.46, 0.91), xaxt ="n",
     main = "B. Consistently good performance group")
lines(x, predict(lo), lwd = 3, lty=1, col = "black")
axis(1, at = 1:10, paste0(seq(90, 900, by = 90)))
### Cubic Splines.
y <- unlist(AUCs[3, ])
lo <- loess(y ~ x)
lines(x, predict(lo), lwd = 3, col = "darkorchid", lty = 1)
### Level Scaling.
y <- unlist(AUCs[5, ])
lo <- loess(y ~ x)
lines(x, predict(lo), lwd = 3, col = "red", lty = 1)
### Pareo Scaling.
y <- unlist(AUCs[9, ])
lo <- loess(y ~ x)
lines(x, predict(lo), lwd = 3, col = "steelblue1", lty = 1)
### Power Scaling.
y <- unlist(AUCs[10, ])
```

```
lo <- loess(y ~ x)

lines(x, predict(lo), lwd = 3, col = "orange", lty = 1)

### Range Scaling.

y <- unlist(AUCs[13, ])

lo <- loess(y ~ x)

lines(x, predict(lo), lwd = 3, col = "springgreen4", lty = 1)

### Vast Scaling.

y <- unlist(AUCs[14, ])

lo <- loess(y ~ x)

lines(x, predict(lo), lwd = 3, col = "yellow3", lty = 1)

### Quantile.

y <- unlist(AUCs[12, ])

lo <- loess(y ~ x)

lines(x, predict(lo), lwd = 3, col = "orange", lty = 2)

### Linear Baseline.

y <- unlist(AUCs[7, ])

lo <- loess(y ~ x)

lines(x, predict(lo), lwd = 3, col = "red", lty = 2)

### Cyclic Loess.

y <- unlist(AUCs[4, ])

lo <- loess(y ~ x)

lines(x, predict(lo), lwd = 3, col = "mediumblue", lty = 2)

### (C) Moderate performance group

### Li-Wong.

y <- unlist(AUCs[6, ])

lo <- loess(y ~ x)

plot(x, y, col = "white", xlab = "Sample size", ylab = "AUC values",
```

```
        ylim = c(0.46, 0.91), xaxt = "n",
        main = "C. Moderate performance group")
lines(x, predict(lo), lwd = 3, lty = 2, col = "springgreen4")
axis(1, at = 1:10, paste0(seq(90, 900, by = 90)))
### PQN.
y <- unlist(AUCs[11, ])
lo <- loess(y ~ x)
lines(x, predict(lo), lwd = 3, col = "darkorchid", lty = 2)
### (D) Poor performance group
### Contrast.
y <- unlist(AUCs[2, ])
lo <- loess(y ~ x)
plot(x, y, col = "white", xlab = "Sample size", ylab = "AUC values",
        ylim = c(0.46, 0.91), xaxt = "n",
        main = "D. Poor performance group")
lines(x, predict(lo), lwd = 3, lty = 2, col = "steelblue1")
axis(1, at = 1:10, paste0(seq(90, 900, by = 90)))
par(op)
```

Source code of program 6: investigating relationship among normalizing methods.

```
### Step 1: Heatmap used in presentation of relationship among methods.
### Heatmap used for investigating the relationships among 15 methods.
### For example, the Euclidean distance and ward.D algorithm was selected.
mydist <- function(x) dist(x, method = "euclidean")
myclust<-function(x) hclust(x, method = "ward.D")
library(d3heatmap)
d3heatmap(AUCs, #    AUCs refer to the matrix consisting of a series of AUC values.
```

```
                    distfun = mydist,
                    hclustfun = myclust,
                    scale="none", colors="RdYlBu", k_row = 4, k_col = 2)
### Step 2: Corrplot (correlation coefficient plot) used in presentation of relationship among methods.
library(corrplot)
data <- read.csv("AUC_matrix", header = TRUE)
sample.data <- data
rownames(sample.data) <- sample.data[, 1]
sample.data <- sample.data[, -1]
op <- par(mfrow = c(1, 1), bg = "white")
M <- cor(t(sample.data))
corrplot(M, method = "ellipse", type = "lower", tl.cex = 0.7, tl.col = "black",
                  rect.col = "black", addCoef.col = "black")
### --------------------------- The end of codes!!! ---------------------------- ###
```
